# Genome editing in cereal crops: an overview

**DOI:** 10.1007/s11248-021-00259-6

**Published:** 2021-07-14

**Authors:** Jerlie Mhay Matres, Julia Hilscher, Akash Datta, Victoria Armario-Nájera, Can Baysal, Wenshu He, Xin Huang, Changfu Zhu, Rana Valizadeh-Kamran, Kurniawan R. Trijatmiko, Teresa Capell, Paul Christou, Eva Stoger, Inez H. Slamet-Loedin

**Affiliations:** 1 Genetic Design and Validation Unit, International Rice Research Institute, Los Banos, Philippines; 2 Department of Applied Genetics and Cell Biology, University of Natural Resources and Life Sciences, Vienna, Austria; 3 Department of Plant Production and Forestry Science, School of Agrifood and Forestry Science and Engineering (ETSEA), University of Lleida-Agrotecnio CERCA Center, Lleida, Spain; 4 ICREA, Catalan Institute for Research and Advanced Studies (ICREA), Barcelona, Spain; 5 Department of Biotechnology, Azarbaijan Shahid Madani University, Tabriz, Iran

**Keywords:** Maize, Rice, Wheat, Barley, CRISPR/Cas9, Talens

## Abstract

Genome-editing technologies offer unprecedented opportunities for crop improvement with superior precision and speed. This review presents an analysis of the current state of genome editing in the major cereal crops- rice, maize, wheat and barley. Genome editing has been used to achieve important agronomic and quality traits in cereals. These include adaptive traits to mitigate the effects of climate change, tolerance to biotic stresses, higher yields, more optimal plant architecture, improved grain quality and nutritional content, and safer products. Not all traits can be achieved through genome editing, and several technical and regulatory challenges need to be overcome for the technology to realize its full potential. Genome editing, however, has already revolutionized cereal crop improvement and is poised to shape future agricultural practices in conjunction with other breeding innovations.

**Supplementary Information:**

The online version contains supplementary material available at 10.1007/s11248-021-00259-6.

## Significance statement

 Modern varieties of cereal crops with higher yields and more resilient to environmental stresses than previous strains have contributed to global food security over the last half century. However, the precision and time needed for the development of new varieties with desirable traits to adapt to climate change and keep up with rapid population growth need to be improved substantially. This review presents an analysis of the current state of genome editing in the major cereal crops rice, maize, wheat and barley. The review thus provides the reader not only with an overview of the latest applications of genome editing for trait improvement in cereals, but also discusses technical limitations and regulatory challenges that need to be overcome for the technology to make an impact in global agriculture.

**Johannes Buyel**, *Fraunhofer Institute for Molecular Biology and Applied Ecology IME, Forckenbeckstrasse 6, 52074 Aachen, Germany*

## Introduction

Rice, wheat, and maize are the three major cereal crops supplying more than 42% of all calories consumed by the global population (Ricepedia 2020). Maintaining a steady supply of these staples while improving their nutritional content and addressing climate change is challenging and requires the application of a number of innovative agriculture breeding strategies. Genome editing is a disruptive technology with profound applications in many sectors including agriculture for crop improvement (Bortesi et al. 2016; Zhu et al. 2017; Zhang et al. 2018; Armario Najera et al. 2019). Genome editing can improve many crops through precise targeted mutagenesis and gene targeting (GT) (Sedeek et al. 2019). Application of genome editing techniques complementing other modern breeding methods can lead to yield gain in a sustainable way.

**Fig. 1:**
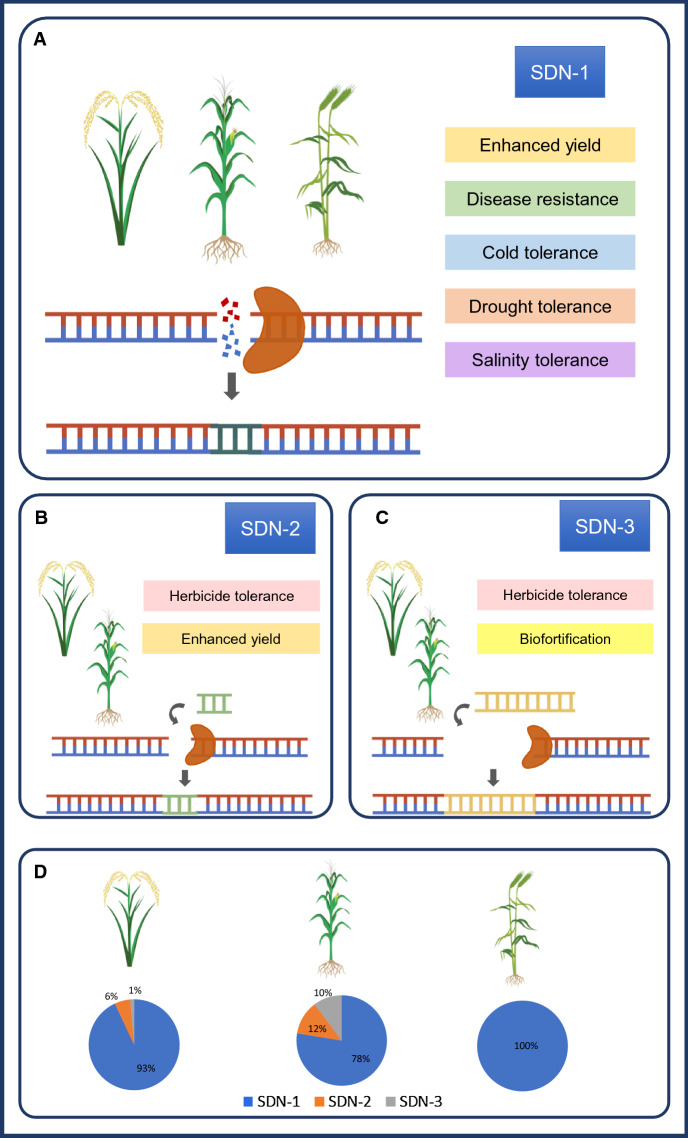
**a** Site directed nuclease (SDN)-1 editing with non-homologous end-joining (NHEJ) DSB repair mechanism and traits developed in rice, maize and wheat. **b** SDN-2 editing, mainly through homology-directed repair and traits developed in rice, maize and wheat. **c** SDN-3 editing, insertion in targeted locus, mainly through homology-directed repair mechanism and traits developed rice, maize and wheat. **d** The current percentage of products developed through SDN-1, 2 and 3 in rice, maize and wheat

**Fig. 2:**
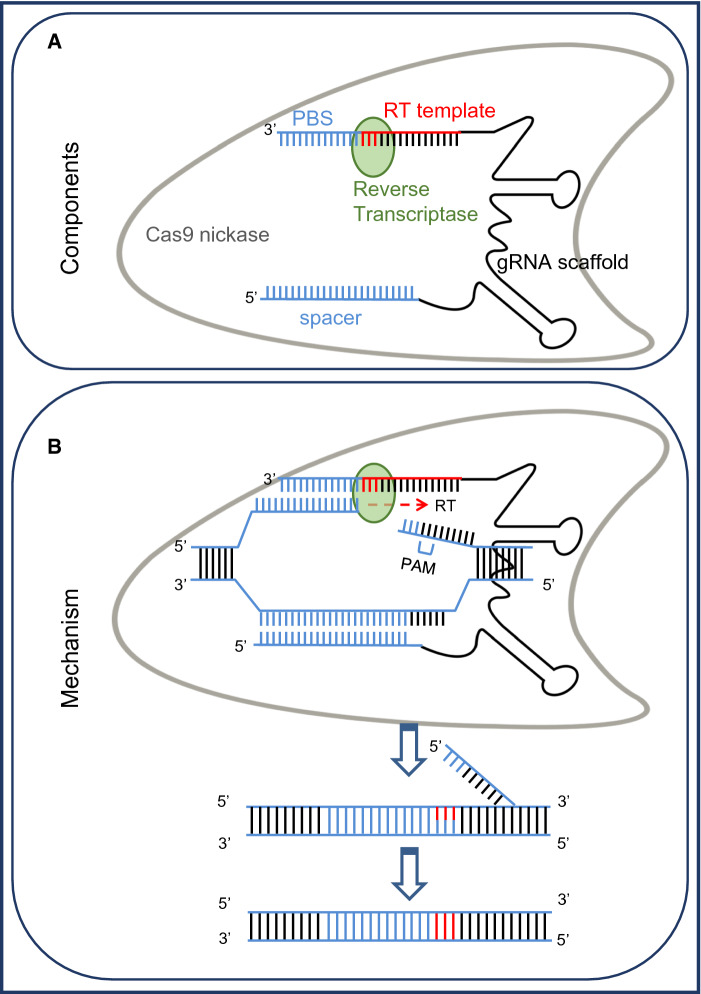
Prime editing components and mechanism. **a** Components of prime editing complex, including a Cas9 nickase fused to a reverse transcriptase and a prime editing guide RNA (pegRNA). The pegRNA is similar to a standard single-guide RNA (sgRNA) but has a reverse transcription (RT) template that contains the desired edit and a primer binding site (PBS) that binds to the target genomic site on the 3′ end. **b** After binding to the target DNA, prime editing complex nicks the PAM-containing strand. The PBS binds to the resulting 3′ end of the nick, and the 3′ end is extended through reverse transcription using the RT template of the pegRNA. The 5′ flap of target DNA is cleaved by cellular endonuclease and the new DNA containing edit is incorporated. The unedited strand is then repaired to match the edited strand

The advancement of a relatively simple editing approach by the Clustered regularly interspaced short palindromic repeats (CRISPR)/Cas system combined with the availability of open-source data of genes and single nucleotide polymorphisms (SNPs) involved in important traits in cereals has resulted in a surge of publications in genome editing for crop improvement. The products of genome editing are often classified as site directed nuclease SDN-1, SDN-2, and SDN-3 (Grohmann et al. 2019). All three mechanisms utilize double-strand break (DSB) repair mechanisms. SDN-1 relies on the error-prone non-homologous end joining (NHEJ) pathway to introduce point mutations at the specific target site resulting in insertion and deletion of a few bases (Fig. 1a). SDN-2 relies on an alternative repair mechanism called homology-directed repair (HDR) and utilizes a template sequence that differs only by a few nucleotides to the existing sequence (Fig. 1b). SDN-3 uses the same mechanism as SDN-2; however, longer DNA sequences are included in the template (Fig. 1c) (Grohmann et al. 2019). Different genome editing techniques have been applied in cereals: Meganucleases, Zinc finger nucleases (ZFNs), Transcription activator-like effector nucleases (TALENs) and CRISPR/Cas9 (reviewed in Zhu et al. 2017). Other than techniques that utilize DSB repair mechanisms, base editors are also used for precise cereal editing. Base editing allows for precise editing at a target site without DSBs or a donor template. Instead, it is based on a fusion of the Cas9 nickase with a DNA deaminase enzyme. *Cytidine deaminases* catalyze conversion of C·G to T·A base pairs while adenosine deaminases catalyze conversion of A·T to G·C base pairs (Komor et al. 2016; Gaudelli et al. 2017; Zhu et al. 2017; Anzalone et al. 2020). A more recent technique is prime-editing, which employs an RNA-programmable nickase fused to reverse transcriptase and a prime editing guide RNA (Fig. 2) (Anzalone et al. 2019). Prime editing offers advantages over CRISPR-Cas9 and base editing because it can create all twelve possible single-base changes, as well as small insertion or deletion mutations (Kantor et al. 2020). Prime editing has been applied in several cereals to develop herbicide-resistant crops (Zhang et al. 2019a, b, c; Butt et al. 2020; Lin et al. 2020b).

This review will discuss the development of important traits in the three principal cereal (rice, maize, and wheat) and the small grain cereal barley, the corresponding genes, and editing technique to introduce or develop the novel traits, future perspectives, and remaining challenges.

## Development of genome editing techniques in cereals

### Maize

Maize was at the forefront of the development of genome editing in cereals, and it is, therefore, useful to consider the different genome editing methods used in this species from a historical perspective before looking at their applications for the modification of agronomic traits. Prior to the development of designer nucleases, targeted mutagenesis was rarely achieved in cereals and the available methods were laborious because they relied on the selection and recovery of extremely rare homologous recombination (HR) events involving an endogenous target and exogenous donor DNA (Cotsaftis and Guiderdoni 2005). An early step toward more efficient GT was the realization that HR is enhanced by the presence of a DSB at the target site, but this created a catch-22 situation in which it was necessary to introduce a DSB at a defined site in order to test whether this would improve the efficiency of targeted transgene insertion by HR. An elegant solution was to introduce the target site for the yeast homing endonuclease I-SceI into the maize genome by standard random integration (D’Halluin et al. 2008). I-SceI recognizes the 18-bp sequence TAGGGATAACAGGGTAAT and leaves a 4-bp overhang. Statistically, this sequence occurs only once in every 70 trillion base pairs and is not naturally found in the maize genome, providing the ideal target site to test the DSB hypothesis. The authors randomly integrated a transgene construct comprising a promoterless *bar* gene downstream of the I-SceI site and attempted to knock in a cassette containing a strong promoter to reactivate the marker and confer herbicide tolerance. HR at the heterologous I-SceI site did not occur in the absence of a functional I-SceI enzyme, but many herbicide-tolerant lines were recovered following the transient expression of a functional enzyme. More than one-third of the recovered lines were knock-in events without random insertions elsewhere in the genome, and some were clean insertions without losses or filler DNA at the knock-in cassette boundaries. The authors did not investigate the effects of I-SceI in the absence of donor DNA because they selected for herbicide resistance dependent on the knock-in cassette, so they did not demonstrate the classic NHEJ-based form of genome editing. This was first reported in maize by Yang et al. (2009), who used the site-specific FLP recombinase to transiently express I-SceI in plants containing a randomly integrated heterologous I-SceI site. The experiment showed that the introduction of DSBs without a knock-in cassette could generate indels at the target site with a frequency of ~ 1%.

The introduction of a heterologous I-SceI site was sufficient to demonstrate the principle of genome editing in maize and to provide so-called safe harbor lines with landing pads optimized for the knock-in of transgenes within active regions of the genome. However, the full power of genome editing could only be realized by targeting endogenous loci. This was achieved in maize by adapting the emerging technologies already demonstrated in mammalian cells and model dicot plants such as Arabidopsis and tobacco, namely ZFNs, engineered meganucleases (EMNs), and TALENs. The first such report involved the targeted disruption of the *INOSITOL PHOSPHOKINASE1* (*IPK1*) locus by the knock-in of a herbicide-tolerance gene using ZFNs, simultaneously conferring a selectable phenotype and reducing inositol phosphate levels in developing seeds, which is a strategy to reduce the levels of phytate (Shukla et al. 2009). The first EMN used in maize was a derivative of I-CreI named LIG3::4, which recognizes a site upstream of the *LIGULELESS1* (*LG1*) gene solely in the genome of the maize inbred EXT (Gao et al. 2010). Although the method used by these authors to modify the target site specificity of the EMN could potentially be used to target any locus, the process is laborious and requires trial and error testing, so the further use of EMNs has been very limited in comparison to other genome editing platforms. A complex system involving dexamethasone-inducible I-SceI and the excision of repair DNA which is used as a template for HR at another target site has also been described (Ayar et al. 2013). The first use of TALENs in maize was a proof-of-concept study to generate stable and heritable mutations at the *GLOSSY2* (*GL2*) locus (Char et al. 2015). Transgenic lines containing monoallelic or biallelic mutations were generated in the Hi-II variety at a frequency of ~ 10%, and a glossy phenotype was confirmed for three of these alleles. In each of these studies, the driver of the gene-targeting event (a transgene encoding the nuclease) was shown to segregate from the mutant locus in the subsequent generations.

Genome editing in maize expanded massively following the development of the CRISPR/Cas9 platform, and the first studies were pioneering because they were also the first to demonstrate multiplex editing (Svitashev et al. 2015) and DNA-free editing via the introduction of Cas9/gRNA ribonucleoproteins (RNPs) (Svitashev et al. 2016). In the first of these studies, five loci were targeted in maize embryos using DNA constructs delivered by particle bombardment, in one case with or without repair cassettes for allele replacement by HR. Mutations were recovered at all five target sites (upstream of *LG1*, in the male fertility genes *MS26* and *MS45*, and in the acetolactate synthase genes *ALS1* and *ALS2*) including multiplex mutations in *LG1*, *MS26,* and *MS45*. Furthermore, allele replacements were recovered in the presence of an *ALS2* donor cassette (Svitashev et al. 2015). In the case of *LG1* and *MS46*, the CRISPR/Cas9 method was tested head-to-head with available EMNs and was found to be at least tenfold more efficient. In the second study, pre-assembled Cas9/gRNA RNPs targeting *LG1*, *ALS2*, *MS26,* and *MS45* were delivered to maize embryo cells by particle bombardment. Again, knock-out mutations affecting all four loci were recovered as well as *ALS2* allele replacements achieved by HR following the co-delivery of a repair template (Svitashev et al. 2016). A contemporaneous study revealed that CRISPR/Cas9 was an efficient tool for genome editing in both the euchromatic and heterochromatic regions of the maize genome (Feng et al. 2016). A more extensive study using a codon-optimized Cas9 gene and optimized gRNA expression showed that the CRISPR/Cas9 system introduced indels efficiently (> 10%) at 90 loci with no off-target mutations and no significant transcriptomic changes (Zhu et al. 2016). The authors also recovered stable lines with knock-out mutations in the *PHYTOENE SYNTHASE1* (*PSY1*) gene which controls the first step in the carotenoid biosynthesis pathway.

Maize was also an early model system for the testing of various CRISPR innovations, including the Cas9 nickase and alternatives to Cas9. For example, Wolter et al. (2017) tested a Cas9 nickase on the target locus *MATERNALLY EXPRESSED IN EMBRYO1* (*MEE1*) to determine the ability of nickases targeting adjacent sites at the same locus or closely-linked loci to generate large deletions. Although the efficiency of the gRNA pairs varied considerably, the authors were able to achieve a high ratio of large deletions to other types of mutation, as well as the insertion of donor DNA fragments. The knock-out of *MEE1* confirmed that transcriptionally inactive and methylated genomic loci could be targeted by Cas9 nickase. Lee et al. (2019b) compared CRISPR/Cas9 to the Cas12a/Cpf1 variant by targeting the *GL2* locus, which includes overlapping sequences recognized by both nucleases. The analysis of on-target mutations showed that 90–100% of the T0 plants generated by Cas9 carried indels (63–77% of which were homozygous or biallelic) compared to only 0–60% of the T0 plants generated by Cas12a. CIRCLE-Seq analysis identified 18 potential off-targets for the Cas9 construct and 67 for the Cas12a construct, each with an average of five mismatches compared to the target site. Sequencing revealed no further mutations in the T1 plants constitutively expressing Cas9 and the corresponding gRNAs.

### Rice

Rice is among the very early crops to be edited and widely studied due to its small genome size, availability of genetic resources and sequence data, and its transformability. In addition, a number of genes and SNPs related to agronomically desirable traits have been studied by comparative genomics, genome-wide association studies (GWAS), and OMICS-based approaches (Endo and Toki 2020). This allows the efficient selection of target genes to be edited. As one of the edited crops, a range of advanced editing techniques has been applied in rice, including base editing and prime editing (Butt et al. 2020; Lin et al. 2020b).

(HR)-mediated GT in rice was initially developed by employing positive–negative selection (PNS) approach (Terada et al. 2002). In this approach, a positive selectable marker is placed in between left and right homology arms, whereas a negative selectable marker is placed upstream of the left homology arms and downstream of the right homology arm. Expression of the negative selectable marker will eliminate cells carrying random integration of T-DNA in their genomes generated via NHEJ pathway. Cells carrying insertion of the positive selectable marker in the targeted position generated via HR pathway will survive in the selection medium. *Waxy* gene was selected as the target gene because screening of the mutants could be conducted by simple iodine staining on pollen and endosperm. The 6.3 kb *Waxy* promoter and 6.8 kb *Waxy* coding regions were used as left and right homology arms, respectively. Using *hpt* gene and a diphtheria toxin (*DT*-*A*) gene as the positive and negative selectable markers, respectively, about 1% of hygromycin resistant calli (6 out of 638) contain the disrupted *waxy* gene as a result of precise HR between the transformed plasmid and the endogenous *Waxy* gene. All of the six fertile transgenic plants had one copy of the *hpt* sequence integrated at the *waxy* locus, in the heterozygous state.

TALENs was used to disrupt rice bacterial blight susceptibility gene *Os11N3* (*OsSWEET14*) (Li et al. 2012). Two pairs of TALENs were employed to induce mutations in two overlapping effector-binding elements (EBEs) within *Os11N3* promoter that are usually bound by the effectors of *Xanthomonas oryzae* to activate the gene and leads to the transport of sucrose out of the leaf cells to meet nutritional needs of the pathogen. Heritable mutations in the targeted sequences were obtained. The effectors delivered by the pathogenic strain of *X*. *oryzae* could not induce the expression of the *Os11N3* gene with mutated EBEs and the plants became resistant.

CRISPR-Cas9 was used to disrupt *OsPDS* and *OsBADH2* genes in rice (Shan et al. 2013). Mutation frequencies of 9.4% (9 of 96 transgenic plants) and 7.1% (7 of 98 transgenic plants) were obtained for *OsPDS* and *OsBADH2*, respectively. Among 9 *OsPDS* mutants 3 showed biallelic mutation and had the albino and dwarf phenotype, confirming the disruption of the rice phytoene desaturase gene. HDR-mediated genome modification of *OsPDS* was achieved in protoplast by co-transformation of Cas9, sgRNA and single-stranded DNA oligos (Shan et al. 2013).

### Wheat

In 2018 a high-quality genome reference sequence for the bread wheat cultivar Chinese Spring was released (IWGSC 2018) which greatly facilitated orientation in the genome. Additionally, reference genomes of the tetraploid species durum wheat and wild emmer, a progenitor of bread wheat, are now available, and a roadmap for the functional characterization of genes in crops with large genomes has recently been published and provides guidance through available genomic tools with a focus on wheat (Adamski et al. 2020 and references therein).

Another challenge with wheat is the recalcitrance of many elite varieties to genetic transformation. To date, delivery of genome editing components has been achieved either by Agrobacterium-mediated or biolistic transformation of immature embryos. Most studies have been carried out with cultivars Bobwhite, Fielder or Kenong 199, but for use in wheat breeding programs the mutated alleles, or the CRISPR/Cas9 transgene, have to be transferred into elite germplasm. Several studies have addressed strategies to improve transformation efficiency per se or to circumvent or develop alternative tissue culture protocols to increase the number of genotypes being amenable to transformation and genome editing. Several tissue culture protocols with improved plant regeneration efficiency have been published for wheat, focusing mostly on the optimization of media compositions and of procedures used for infection with Agrobacterium (Ishida et al. 2015; Hayta et al. 2019). These studies report improved efficiency for Agrobacterium-mediated transformation, however, although they may increase efficiency also in elite varieties, their primary contribution relates to varieties already used in transformation studies, while the applicability of these protocols to the transformation of elite varieties remains to be evaluated. Furthermore, since tissue culture protocols are not easily transferable between labs, reported efficiencies are not always reproducible (Hayta et al. 2019). Co-expression of morphogenetic regulators has been shown to increase regeneration efficiency in monocots. For example, Baby-boom and Wuschel help in recovering healthy, fertile T0 maize and sorghum plants (Lowe et al. 2016). A more recent study reports that the co-expression of a chimeric gene including wheat *GROWTH-REGULATING FACTOR* (*GRF*) and its cofactor *GRF-INTERACTING FACTOR* (*GIF*) improves transformation efficiency in wheat, triticale and rice and could be successfully applied even to recalcitrant wheat genotypes (Debernardi et al. 2020). Another study using dicots and maize described a similar effect upon co-expression of maize GRF5 alone (Kong et al. 2020).

Double-haploid induction is routinely used in crop breeding to accelerate the generation of inbred lines with desired genotypes. Explants containing haploid cells, such as microspores or anthers, are cultured in vitro and subsequently doubling of the genome is chemically triggered or may occur spontaneously (Kalinowska et al. 2019). In barley, this approach together with co-cultivation of *Agrobacterium* containing Cas9/gRNA constructs has been shown to generate edited plants, also in a variety of commercial elite cultivars (Han et al. 2020). In wheat, the cultivars Bobwhite and AC Nanda, which are highly responsive to stress-induced microspore embryogenesis, were genome edited by delivery of Cas9/gRNA via microspore electroporation (Bhowmik et al. 2018).

To avoid the transformation step altogether in wheat, genome editing has been shown to be achievable by intergeneric pollination of several bread wheat and durum wheat cultivars with maize, acting as the transgenic Cas9/gRNA pollen donor (Kelliher et al. 2019; Budhagatapalli et al. 2020). As a result of the wide cross, the maize chromosomes are eliminated after zygote formation in the course of initial embryonic cell divisions and the resulting haploid wheat plants can be diploidized by colchicine treatment (Budhagatapalli et al. 2020). Diploid progenies in two different cultivar backgrounds were generated with homozygous mutant state of one of the targeted homeologs and the plants showed the expected phenotype of reduced plant height. A pollen-specific promoter was used to boost Cas9 expression at the critical time for wide crosses (Kelliher et al. 2019).

Genome editing without in vitro culture and regeneration was also achieved by in planta particle bombardment (Hamada et al. 2018). Imbibed mature seed embryos were bombarded and plants grown from the seeds passed on the edited sites to their progeny (three independent T1 progenies of 210 bombarded plants). Genome editing was carried out in Bobwhite, however, the in planta transformation method had been shown earlier also to work in the commercial elite cultivar ‘Haruyokoi’ and in the experimental cultivar Fielder (Hamada et al. 2017). Viral vectors may also provide a solution to circumvent tissue culture recalcitrance: barley stripe mosaic virus-based gRNA delivery in a transgenic wheat line harboring Cas9 was able to induce editing at the desired target site (Hu et al. 2019b).

Finally, there is always the possibility to generate edited lines of a genotype amenable to transformation and then cross them with elite cultivars to transfer the mutations and resulting traits. However, the targeted locus may sometimes not be present in transformable genotypes and secondly, there might be an undesirable linkage drag, depending on the neighboring regions and the crossing over frequency in the targeted region. To circumvent this effect, elite cultivars may be crossed with cultivars harboring the Cas9/gRNA cassette and F1 individuals are subsequently back-crossed to eliminate the Cas9/gRNA donor genome. It has been shown that this is sensible for wheat as gRNA targets that are not yet modified in early generation plants can be edited in the following generations (Wang et al. 2018a).

The various published and proposed strategies for editing elite genotypes may bring additional advantages to the process of genome editing, for example doubled haploid induction, but also require meticulous adoption to be implemented successfully in a laboratory. It remains to be seen which of these strategies will be adopted for efficient routine genome editing in elite cultivars.

Wheat transformation in general is a lengthy and still in many cases inefficient process, therefore in the context of genome editing sgRNAs are often tested for efficiency in wheat protoplasts before proceeding to establish edited lines. Reported editing efficiencies (edited T0 plants per transgenic T0 plants) of gRNAs range from 5 to 17% (Howells et al. 2018) to 37% (eight independent transgenic T0 plants from 342 explants, of which three were edited based on PCR-RE analysis) (Abe et al. 2019). In some studies, genotyping for editing was started in the T1 generation: edited offspring from all 4 transgenic T0 individuals were reported in a study targeting NFXL1 (Cui et al. 2019), and 25 out of 181 T1 plants with 32 independent mutations, originating from 47 primary T0 plants were reported in a study targeting Ms45 (Singh et al. 2018).

Newest developments in CRISPR-Cas based editing have been applied to wheat. Most recently, prime editing programmed to establish nucleotide substitutions, was tested in wheat protoplasts with a reported frequency of 1.4% (counted by amplicon deep sequencing) (Lin et al. 2020b). Base editing by adenine deaminase (Li et al. 2018c) and cytidine deaminase fused to Cas9-nickase (Zong et al. 2017; Zhang et al. 2019a) were used to create herbicide tolerant wheat plants (Zhang et al. 2019b). In the latter study the authors demonstrated that base editing at a specific ALS site confers resistance to nicosulfurin herbicide and propose a selectable co-editing marker system based on the ALS target. Furthermore, a system generating predictable multi-nucleotide-targeted deletions within the protospacer was developed based on a fusion of fully functional Cas9 and cytidine deaminase (APOBEC–Cas9 fusion-induced deletion systems (AFIDs)), resulting in a deletion ranging from the deaminated cytosine to the DSB initiated by Cas9 (Wang et al. 2020d).

A replicon-based system of a deconstructed version of the wheat dwarf virus (WDV) has been tested for GT in wheat (Gil-Humanes et al. 2017). A fluorescent reporter was placed into the third exon of a ubiquitin locus by GT using Cas9 in wheat protoplasts, and this was achieved at a frequency of 3,8%, as estimated by flow cytometry. A similar frequency was estimated by transformation of scutellum tissue. A second study addressing GT used a ZFN directed to the acetohydroxyacid synthase (AHAS) gene in combination with a repair template delivered by particle bombardment to induce herbicide resistance (Ran et al. 2018).

Due to regulatory (and other) reasons, DNA free delivery of editing components into plants has been investigated. Genome editing at targeted sites was achieved by coating of Cas9/sgRNA ribonucleoprotein complexes or RNA encoding Cas9 and sgRNA onto microcarrier beads for particle bombardment, and subsequent regeneration of explants without selection (Zhang et al. 2016; Liang et al. 2017). A notable difference between using ribonucleoprotein bombardment versus DNA bombardment was lower off-target activity in the former. Very recently, DNA-free delivery of editing components was accomplished by using cell-penetrating peptides complexed with a ZFN for transfection into wheat microspores or embryo like structures (Bilichak et al. 2020).

### Barley

With its sequenced and well annotated diploid genome and its amenability to transformation techniques and in vitro culture, self-pollinating barley may be considered as a diploid model species for closely related hexaploid cereals. Barley is the fourth most abundant cereal grown. The majority of the production is processed for animal feed, and only a minor part goes to human consumption where the main use is for alcoholic beverages. The haploid genome of barley is ~ 5,3 Gb in size spread across seven chromosomes. In 2012 the International Barley Genome Sequencing Consortium published the first reference genome derived from the cultivar Morex (Mayer et al. 2012) which has since been improved in sequence depth, genome assembly and annotation (Mascher et al. 2017; Monat et al. 2019) and can be accessed via http://barleysequence.org/. A recent overview on barley and its role as a model species can be found in (Rotasperti et al. 2020).

Barley transformation is mostly carried out via immature embryo explants and transformation efficiency is dependent on the genotype (Kumlehn and Hensel 2009). The cultivar “Golden Promise” is one of the most efficiently transformable genotypes and recently a reference genome assembly was published for this genotype (Schreiber et al. 2020). To date, genome editing in barley has been mostly carried out in the cultivar “Golden Promise”, but as with other crops there is an increasing need to apply genome editing also directly to current elite cultivars. Gerasimova et al. (2018) showed that this is possible in principle by modifying tissue culture protocols for cultivating protoplasts derived from a Siberian barley cultivar. Successful editing of five local cultivars from Kazakhstan was also reported in an abstract sent to The European Biotechnology Congress 2019 (Kershanskaya et al. 2019). Recently, genome editing was shown in T0 individuals of four commercial cultivars from Australia, which were transformed using agrobacterium-mediated delivery of CRISPR/Cas9 constructs into anther cultures (Han et al. 2020).

An early effort to establish genome editing in barley involved TALENs and did not target a coding region, but the promoter region of the phytase *HvPAPhy-a* (Wendt et al. 2013). The study suggested that barley was amenable to editing without the need to generate a vast number of primary transformants, as on average one out of four plants carrying the selection marker showed editing activity. Subsequent genome editing studies, in which targeted double strand breaks were mainly introduced by agrobacterium-mediated delivery of the classical Cas9 system, confirm that editing efficiency is not a bottleneck. Reported editing efficiencies, that is number of edited T0 per transgenic T0 individuals (as determined by the presence of the Cas9 transgene or selection marker), range from 13 and 25% (Yang et al. 2020) to 46% (Vlčko and Ohnoutková 2020), 78% (Kapusi et al. 2017) and even 88% (Gasparis et al. 2018). In some cases, editing events are screened by methods other than sequencing, and therefore reported efficiencies may be conservative estimates. It is important to note that chimerism is frequently observed in a primary transformant, and several different mutant alleles may be identified within the same plant (Kapusi et al. 2017; Han et al. 2020; Zeng et al 2020a). Analyzing several primary transformants and their offspring stemming from the same transformation event may therefore identify individuals with a distinct mutation pattern and increase the chance to find individuals with the desired mutant sequence.

It is desirable to isolate homozygous mutant plants as early as possible and preferably without the Cas9 transgene. This is feasible in the T1 generation in barley as shown in several studies (Kapusi et al. 2017; Gasparis et al. 2018; Zeng et al. 2020a). Gasparis et al. (2018) followed up ten T1 individuals from each of 22 isolated T0 mutants and characterized them with respect to mutant status and presence of Cas9. They identified twenty Cas9 free mutants, and two of them were homozygous. Another strategy to fix genotypes in a homozygous state within a short time is doubled haploid (DH) production which has been widely used in barley breeding (Broughton et al. 2014). To this end, isolated microspores (Gurushidze et al. 2014) or anthers (Han et al. 2020) are cultured to obtain haploid plants which subsequently undergo induced or spontaneous chromosome doubling. Gurushidze et al. (2014) used embryogenic pollen derived from a barley plant carrying a GFP-transgene to introduce a TALEN pair directed against GFP and detected homozygous mutants in approximately 20% of selection marker positive regenerated plants. Alternatively, T0 individuals with desired edits can be selected for microspore isolation and DH production (Conorado et al. 2005; Kapusi and Stöger 2018). DH production may be particularly useful when targeting several unlinked genes and consequently finding homozygous individuals of higher order mutants is elaborate and time consuming.

To date there is one study in barley demonstrating GT, i.e. using homology derived repair to introduce a priori defined changes via providing a repair template. To this end, a TALEN pair was co-bombarded together with a repair template into barley leaves expressing GFP, and the repair template induced the conversion of GFP to YFP in about 3% of mutated cells (Budhagatapalli et al. 2015).

There are also some examples of gene editing by DSB in barley, which did not result in knock-out mutants. As mentioned above, one of the first editing studies targeted a HvPAPhy-a promoter region containing known TF motifs (Wendt et al. 2013; Holme et al. 2017), and in the course of the study additional promoter regions were identified, which had not been functionally annotated before. Creating fragment deletions in the ENGase gene led in one case to an allele encoding for an internally truncated protein with otherwise maintained N- and C-terminal regions (Kapusi et al. 2017). Although this was not the aim of the study and the presence of the modified protein was not confirmed, this shows the possibility of creating alleles with more subtle changes. Exactly this was the goal when re-creating the *albostrian* mutation in barley (Li et al. 2019a). *Albostrians* is a mutant with variegated leaves due to chloroplast malfunction. It was discovered in the 50ies after X-ray irradiation and was instrumental in studying retrograde signaling. TILLING mutants confirmed the identity of the *albostrians* gene as *HvCMF7* (Li et al. 2019a), however, they showed a more severe phenotype than original *albostrians* plants. In the original *albostrians* mutant a deletion of four nucleotides leads to a C-terminally truncated protein. Using CRISPR/Cas-based editing, a region close to the original deletion was targeted, and an individual with a one base-pair insertion at the expected DSB site reproduced an *albostrians* phenotype (Li et al. 2019a).

## Application of genome editing in trait improvement

### Agronomic traits

#### Maize

Although many of the studies in maize described above involved the use of herbicide-tolerance marker genes, most were proof-of-principle experiments to test genome editing efficiency rather than attempts to modify agronomic traits. However, having established the technology platform and confirmed its efficiency for single and multiple GT, many subsequent studies considered how genome editing could be exploited to improve traits such as biotic and abiotic stress resistance and intrinsic yield potential (summarized in supplementary Table 1). This has involved not only knock-out mutations but also various different knock-in and allele replacement strategies, including the replacement of promoters to boost endogenous gene expression. For example, transgenic plants expressing *ARGOS8* achieve higher grain yields because ARGOS8 is a negative regulator of ethylene responses. The low endogenous expression of *ARGOS8* was increased by either inserting the maize *GOS2* promoter immediately upstream of the *ARGOS8* gene (knock-in) or replacing the native *ARGOS8* promoter with the stronger *GOS2* promoter (allele replacement). The resulting plants achieved higher yields under flowering stress and showed no yield losses under drought conditions (Shi et al. 2017).

Maize plants with upright leaves can be planted at higher densities without shading, which increases the grain yield per unit area (Brekke et al. 2011). Leaf inclination in maize is controlled by the ligule and auricle, structures located at the hinge of the sheath and blade that allow the leaf to project at an angle from the culm. Liguleless mutants lack these structures and field experiments with liguleless hybrids showed a potential for higher grain yields (Lambert and Johnson 1978). The *LG1* gene is strongly associated with the upper leaf angle (Tian et al. 2011) and was, as discussed above, an early target for proof-of-principle genome editing experiments in maize, including EMNs (Gao et al. 2010) and CRISPR/Cas9 (Svitashev et al. 2015) although plant phenotypes were not reported. However, the liguleless phenotype was confirmed in *lg1* knock-out lines produced using the SpCas system, indicating that genome editing can be used to increase planting density (Li et al. 2017a). Even so, higher planting density can lead to other issues such as increased lodging and shading, which can counteract the yield gains. The analysis of a quantitative trait locus (QTL) that improves stalk strength revealed the *STIFF1* gene (encoding an F-box protein that inhibits cellulose and lignin synthesis) and the presence of an internal transposable element that represses its expression and therefore produces stronger stalks (Zhang et al. 2020c). This effect was replicated by the authors using the CRISPR/Cas9 system to create a heterozygous mutant also with increased lodging resistance (Zhang et al. 2020c). Knocking out the *PHYC1* and *PHYC2* genes encoding red and far red photoreceptor phytochromes eliminated the shade avoidance syndrome that reduces yield in crowded plots. Double knock-out mutants generated using the CRISPR/Cas9 system showed a precocious flowering phenotype under long-day (LD) conditions indicating that maize photoreceptor phytochromes act as floral repressors during long days (Li et al. 2020b). Similarly, using CRISPR/Cas9 to knock-out the *CCT9* gene caused early flowering under LD conditions, also revealing that a natural *Harbinger*-like transposable element located 57 kb upstream the gene functions as a natural repressor (Huang et al. 2017).

Yield optimization can also be achieved by the manipulation of developmental signalling pathways including hormone transport and signal transduction and the activity of second messengers and heterotrimeric G proteins that regulate shoot development in response to signals from cell-surface receptors (Gomes et al. 2016). Several studies have addressed the effects of mutations in G proteins but the impact on growth and yield has been detrimental. Knocking out the G protein beta subunit gene triggers lethal autoimmunity (Wu et al. 2019), and triple mutants inactivating all three extra-large GTP binding proteins, which are non-canonical proteins containing a G protein alpha domain, showed a striking developmental arrest and died at the seedling stage (Wu et al. 2018). The analysis of hormone biosynthesis, transport and signalling has achieved more promising results. Knocking out the gibberellin-oxidase20-3 gene blocked the gibberellin biosynthesis pathway and generated semi dwarf maize plants with increased lodging resistance (Zhang et al. 2020b). Similarly, using CRISPR/Cas9 to target the *BRACHYTIC2* (*BR2*) gene, which encodes an ATP binding cassette type B (ABCB) involved in auxin transport, induced a 1-bp frameshift that generated a premature stop codon in exon 5 and resulted in a semi dwarf phenotype similar to natural *br2* mutations.

#### Rice

Currently, rice consumers globally reach more than 3 billion people, and rice is related to not only food security but also economic growth, culture, and regional stability (Yadav and Kumar 2018). Accelerating genetic gain to cover the demand of rice supply is one of a major challenges. The integration of novel technologies into rice breeding programs is essential to increase agricultural productivity when aligned with principles of quantitative and Mendelian genetics (Cobb et al. 2019). Many reports demonstrated the successful application of innovative breeding genome editing technologies for rice improvement ((summarized in supplementary Table 2).

An *indica* hybrid rice cultivar Guang Liang You 1128 (GLY1128) is known for its excellent agronomic traits. Despite this, it has a strong seed shattering phenotype. In hopes of rectifying this issue, targeted mutagenesis of *qSH1* gene was performed on its parental lines, HR1128 and Guangzhan63-4S, using a CRISPR/Cas9 vector expressing two gRNAs targeting sense and anti-sense strands of sequences containing the start codon of the gene. T_2_ *qSH1* mutant lines expressing truncated and altered qSH1 proteins exhibited significantly reduced seed shattering (69% increase in breaking tensile strength). No significant differences were observed on major agronomic traits between mutants and WT. Crossing between mutant HR1128 and mutant Guangzhan63-4S generated mutant hybrid rice line with improved seed shattering phenotype (57% increase in breaking tensile strength) with no significant morphological differences or changes in grain yield relative to the control hybrid rice line (Sheng et al. 2020).

Rapid and uniform germination of seeds is important for rice production. After harvesting, rice seeds are usually dried under sunlight and incubated at 37–55 °C for a few days to break the dormancy. Targeted mutagenesis of rice *viviparous-1* (*OsVP1*), a homolog of *Arabidopsis ABSCISIC ACID INSENSITIVE 3* (*ABI3*), was performed on rice cultivar Dongjin using a CRISPR/Cas9 vector expressing a gRNA targeting the first exon of *OsVP1* gene. The mutant seeds began to germinate 1 day after sowing, whereas WT seeds started to germinate 2 days after sowing. Under normal cultivation conditions, the mutant lines did not show significant differences in the main agronomic traits (Jung et al. 2019a).

The architecture of a rice plant (the structure and arrangement of organs) affects many important agricultural traits, including the grain yield. The root system architecture is important for stability, as well as influencing hormone biosynthesis and the absorption of nutrients and water (Coudert et al. 2010). The shoot architecture is important for photosynthetic efficiency, particularly the angle between the leaf and culm (Sinclair and Sheehy 1999; Wang and Li 2011). Plants with upright leaves allow more light to reach lower leaves, thus optimizing canopy photosynthesis at higher field densities (Sinclair and Sheehy 1999). All of these factors affect growth, and therefore influence the yield. One of the most important architectural traits in rice is branching, which includes tillering (the formation of additional stems on the basal node) and panicle branching (which increases the number of grain-bearing structures), both contributing to the number of grains produced per plant (Wang and Li 2011). Grain yield not only depends on the grain number, but also the grain size, shape and weight (Xing and Zhang 2010). The transcription factor NAC2 regulates grain yield in rice by suppressing root growth and thus the ability of plants to take nutrients from the soil. The CRISPR/Cas9 system has been used to knock-out the rice *NAC2* gene, resulting in plants with longer primary roots and more crown roots than WT plants, also increasing the sensitivity of the roots to auxins and cytokinins (Mao et al. 2020). Previously, the same group had shown that the suppression of *NAC2* by RNA interference (RNAi) led to a ~ 10% increase in the grain yield (Mao et al. 2017). These data suggest that the knock-out of *NAC2* is a potentially useful strategy for yield enhancement. Conversely, the knock-out of *ABA1* using the CRISPR/Cas9 system resulted in a short root phenotype and slender plants (Lin et al. 2020a). Furthermore, all the mutant lines died in the field without seed setting, confirming that ABA is necessary for plants to complete their life cycle under ambient conditions.

The role of auxins in root development has been investigated by multiplex genome editing, which allows the simultaneous mutation of multiple genes in the same family or even in different families (Armario Najera et al. 2019). Auxin levels are determined by the activity of auxin efflux carrier proteins of the PIN-formed family (PIN). There are four *PIN1* homologs in rice (*PIN1a–1d*) and multiplex genome editing with the CRISPR/Cas9 system has been used to determine their individual functions and the potential for functional redundancy (Li et al. 2019b). Compared with the WT, the *OsPIN1* single mutants did not show dramatic phenotypes, suggesting its function is mostly compensated by other paralogs. In contrast, the *pin1a pin1b* double mutant featured shorter shoots and primary roots, fewer crown roots, reduced root gravitropism, longer root hairs and a larger panicle branch angle. The *pin1c pin1d* double mutant showed no observable phenotype at the seedling stage, but produced naked, pin-like inflorescences at the flowering stage. These data suggest that *PIN1*a and *PIN1b* are involved in root, shoot and inflorescence development, whereas *PIN1c* and *PIN1d* mainly function in panicle formation (Li et al. 2019b).

Leaf inclination in plants is influenced by genetics, hormones and nutritional status, in particular the availability of inorganic phosphate (Pi). Ruan et al. (2018) found that Pi deficiency repressed the expression of *RLI1* (*REGULATOR OF LEAF INCLINATION 1*), which induces leaf inclination. RLI1 is a transcription factor that triggers lamina joint cell elongation by binding to the promoter element NNAKATNC to regulate the transcription of *BU1* (*BRASSINISTEROID UPREGULATED 1*) and *BC1* (*BU1-LIKE1 COMPLEX 1*)*.* However, Pi deficiency stress activates the Syg1/Pho81/XPR1 family proteins SPX1 and SPX2, which suppress inclination by interacting directly with RLI1. Ruan et al. (2018) used the CRISPR/Cas9 system to knock-out the *BU1* and *BC1* genes in WT plants and a transgenic line overexpressing *RLI1*, the latter with significantly inclined leaves. The exaggerated leaf inclination in the transgenic lines was suppressed by the *bu1* or *bc1* mutations, confirming that RLI1 acts upstream of BU1 and BC1 (Ruan et al. 2018). Genome editing has been used to analyze the functions of many other candidate genes that control architectural traits, including those involved in root or shoot architecture and photosynthetic efficiency.

The *GA20ox2* gene encoding gibberellin oxidase contributes to the “Green Revolution” semi-dwarf phenotype in rice, and this *sd1* mutation has been recreated using the CRISPR/Cas9 system (Han et al. 2019). Exogenous gibberellin restored the normal height to the mutant plant, confirming that the stunting is caused by gibberellin deficiency. Accordingly, the gibberellin content was lower than WT levels with a commensurate 22% reduction in plant height, a longer flag leaf length and a 6% improvement in yields, with no effect on other agronomic traits (Han et al. 2019). The *sd1* mutation has also been introduced into the elite landraces Kasalath and TeTePu (TTP), which feature many desirable agronomic traits such as resistance to biotic and abiotic stress and tolerance of low Pi levels (Hu et al. 2019a). As expected, the mutants were stunted and showed better lodging resistance than WT controls, and the yields were higher in field trials. However, the effect of *sd1* alleles generated by CRISPR/Cas9 depends on the genetic background, and in some cases can reduce rather than improve yields (Biswas et al. 2020).

Homozygous mutants of *SRL1* (*SEMI-ROLLED LEAF1*) and *SRL2* created using the CRISPR/Cas9 system featured a curled leaf phenotype and showed improved drought tolerance (Liao et al. 2019). Hybrids generated from mutant restorers showed a semi-rolled leaf phenotype and produced higher yields due to the formation of more panicles and more grains per panicle (Liao et al. 2019).

A regulatory loop that integrates the circadian clock, sugar accumulation and the strigolactone pathway to regulate tiller-bud and panicle development has been investigated using the CRISPR/Cas9 system (Wang et al. 2020b). The *CCA1* (*CIRCADIAN CLOCK ASSOCIATED 1*) gene encodes a negative regulator of tillering, so overexpressing this gene reduces the tiller number and knock-out mutants produce more tillers. In contrast, *PRR1* (*PSEUDORESPONSE REGULATOR 1*) encodes a positive regulator, so overexpressing this gene increases the tiller number and knock-out mutants produce fewer tillers. CCA1 directly regulates *IPA1* expression to control panicle and grain development (Wang et al. 2020b). Overexpression and knock-out lines for each of these genes were used to investigate the functions of *SCM3 (STRONG CULM 3)/TB1* (*TEOSINTE BRANCHED 1*), *D14* (*DWARF 14*) and *IPA1*, revealing that all three act downstream of *CCA1*. Sugars repress *CCA1* expression in roots and tiller buds to promote tiller-bud outgrowth. The circadian clock integrated sugar responses and the strigolactone pathway to regulate tiller and panicle development, providing a potential new target for the improvement of rice yields (Wang et al. 2020b). CRISPR/Cas9 has also been used to introduce mutations scanning along the promoter and 5′ untranslated region of the *SCM3/TB1* gene in variety Nipponbare, resulting in three categories of mutants with lower or abolished expression (type 1), unaffected expression (type 2) or enhanced expression (type 3) of *SCM3/TB1* (Cui et al. 2020). The type 1 mutants showed a range of beneficial phenotypes, including additional tillers and smaller culms and panicles (Cui et al. 2020) resembling the *TB1* null mutant *fc1-2* (Minakuchi et al. 2010). In contrast, the type 2 mutants were similar to WT plants and the type 3 mutants had fewer tillers and larger culms and panicles, mimicking the phenotypes of varieties that overexpress *TB1*, such as NILSCM3 and 93–11 (Yano et al. 2015). The CRISPR/Cas9 system can therefore be used to generate allelic series by editing *cis*-regulatory elements as an additional strategy to improve the architectural traits of rice plants.

#### Wheat

Among the big five of the crop monocot genomes (Haberer et al. 2016) bread wheat ranks as number one with 17 Gb spread over 21 chromosomes. Due to this large genome size and its hexaploid composition of three subgenomes, the A, B and D genome, breeding as well as genome editing are challenging in this species. For example, barley plants with loss of function alleles at a single locus, *MILDEW RESISTANCE LOCUS* (*MLO*), exhibit strong, broad spectrum resistance against the powdery mildew fungus; to achieve the same effect in wheat, the three homeologs of *MLO* present in the three subgenomes had to be edited simultaneously. Nevertheless, wheat was one of the first crop species modified by genome editing, and powdery mildew resistance via TALEN-mediated knock-out of *MLO* is one of the early success reports (Wang et al. 2014). Improvements of important traits via genome editing in wheat are summarized in supplementary Table 3.

Yield, in relation to grain morphology and weight was modulated by targeting *TaGW2* (Wang et al. 2018b) and *TaGW7* (Wang et al. 2019a). Editing the TaGW7 B and D homeologs led to increased grain width and weight but reduced grain length, while seeds of a triple mutant in *TaGW2* have increased thousand-grain weight (TGW), grain area, width and length in the cultivar “Bobwhite”. The contribution of each of the *TaGW2* homeologs to the quantitative trait was investigated in detail, and dosage dependent additive effects of the homeologs were found (Wang et al. 2018a, b; Wang et al. 2019a, b, c, d). When comparing the *TaGW2* editing mutants to mutants derived from TILLING in the cultivar “Paragon”, inter-cultivar differences were found with respect to the contribution of the homeologs to the yield traits (Wang et al. 2018b). In general, the additive effects observed across homeologs and genotypes can be correlated with their level of gene expression, which may vary between cultivars.

Seed morphology and yield are influenced by plant hormones and for example the inhibition of cytokinin degradation by Cytokinin oxidase/dehydrogenase (CKX) has been shown to result in higher cytokinin accumulation and a higher number of reproductive organs. Several reports have demonstrated that grain yield can be improved by the suppression of CKX expression. For example, the downregulation of TaCKX2‐D1 significantly increased the grain number per spikelet in wheat (Zhang et al. 2019a).

Seed dormancy is another important agronomic trait. Seeds must not show pre-harvest sprouting, but a regular germination behavior upon sowing is expected. In its recessive state the gene underlying the barley QTL Qsd1 leads to longer dormancy and prevents pre-harvest sprouting. Homeologs for Qsd1 were identified in wheat and its three homeologs were targeted near the site of the natural mutation found in the barley recessive mutant (Abe et al. 2019). To obtain Cas9 transgene free individuals, the authors included a back-crossing step of a T0 aaBbdd wheat plant with the WT cultivar Fielder and selected in the F1 heterozygous mutant individuals without Cas9. Cas9 free individuals with all combinations of homozygous mutants were obtained in F2. Embryo rescue was used to speed up the process, which was carried out in 14 months. Homozygous mutants in all three homeologs (aabbdd) germinated with a delay of about 5 days, whereas all other mutant combinations did not show a statistically significant difference.

CRISPR/Cas9 mutagenesis was also used to create a single mutant in *Ms1* (Okada et al. 2019) and a triple mutant in *Ms45* (Singh et al. 2018) which are monocot nuclear male fertility genes. Homozygous individuals were shown to be male sterile. Following gene cloning, the original *ms1* mutant was previously complemented with one wildtype copy and the *ms45* triple mutant could be complemented with a WT copy of *Ms45* from rice. These findings are prerequisites for the possible use of these loci in seed production technology (Wu et al. 2016; Tucker et al. 2017). Therefore, genome editing may serve as an important tool for the implementation of nuclear male sterile lines for hybrid seed production in wheat. In contrast to maize and rice, hybrid breeding is currently only a small sector in commercial wheat production (Mette et al. 2015). *Ms26*, a third possible gene for inducing nuclear male sterility is currently under investigation using CRISPR/Cas9 (Cigan et al. 2017).

Plant hormones control a number of important physiological processes which in turn determine agronomic traits. Editing genes involved in plant hormone signaling is therefore an interesting approach. For example, the first published gene editing study using Cas9 targeted two *HvPM19* barley gene copies which are ABA regulated plasma membrane proteins. Two homologs in wheat are strong candidate genes for a major and widely deployed QTL in breeding for seed dormancy (Lawrenson et al. 2015, and reference therein). Cytokinin oxidase/dehydrogenase (CKX) degrades the phytohormone cytokinin and reduction of CKX2 is expected to impact on grain yield by causing higher number of reproductive organs via cytokinin accumulation (Holubová et al. 2018). Several reports have demonstrated that grain yield can be improved by the suppression of CKX expression. For example, the downregulation of the CKX genes *Gn1a-2* and *Gn1a-10* by genome editing in rice led to a higher number of flowers per panicle (Li et al. 2016). The use of CRISPR/Cas9 to knock-out the barley cytokinin oxidase/dehydrogenase genes *CKX1* and *CKX3* revealed a loss of CKX enzyme activity in the ckx1 knock-out but no change in the yield of either mutant (Gasparis et al. 2019).

### Disease resistance

#### Rice

Bacterial blight disease, caused by *Xanthomonas oryzae* pv. *oryzae (Xoo),* is a major rice disease that affects rice production. *Xoo* produces transcription activator-like effectors (TALEs) which induce expression of *SWEET* genes by binding to the effector binding elements (EBEs) in the promoter region to establish host susceptibility (Zaka et al. 2018). Three genes that belong to clade III of the *SWEET* gene family were reported as *Xoo* susceptibility (*S*) genes (Streubel et al. 2013). Known major TALEs are PthXo1 that induces *OsSWEET11* (Antony et al. 2010), PthXo2 that induces *OsSWEET13* (Zhou et al. 2015), and PthXo3, AvrXa7, TalC and TalF that induce *OsSWEET14* (Antony et al. 2010; Yu, et al. 2011; Streubel et al. 2013). In addition, two other PthXo2 variants, PthXo2B and PthXo2C were identified in Asian *Xoo* strains capable of inducing *OsSWEET13* in *japonica* reference line Kitaake that is resistant to PthXo2-dependent *Xoo* strains (Oliva et al. 2019).

The EBEs present in the promoter regions of the *SWEET* genes have been the target of genome-editing technologies to create heritable modifications in the genome that will disable *S* gene activation, thus providing a counter defense to the *Xoo* infection strategy. TALEN-induced mutations on overlapping effector binding elements for PthXo3/AvrXa7 in the promoter of *OsSWEET14* were shown to confer resistance against bacterial blight (Li et al. 2012; Blanvillain-Baufume et al. 2017). Similarly, CRISPR/Cas9 edited plants carrying mutations in the EBE of *OsSWEET13* disabled PthXo2-based disease susceptibility (Zhou et al. 2015). In another strategy, naturally-occurring resistant alleles, in combination with mutant alleles induced by CRISPR/Cas9, were examined for broad-spectrum resistance against the disease. The EBEs of *OsSWEET11* and *OsSWEET14* were edited in *japonica* reference line Kitaake, which contains a naturally occurring resistant allele of *OsSWEET13,* resulting in a mutant line exhibiting resistance to the majority of the *Xoo* strains tested (Xu et al. 2019). This combination of *SWEET* promoter variants is a promising approach to develop durable broad-spectrum resistance against bacterial blight. In the study of Oliva et al. (2019), CRISPR/Cas9 multiplex strategy was used to develop a mutant Kitaake rice line, and mutant lines of mega-varieties IR-64 and Ciherang-Sub1 with different combinations of mutations in the EBEs of *OsSWEET11*, *OsSWEET13*, and *OsSWEET14* promoters conferred resistance to a large number of *Xoo* strains tested. In several cases, a compatible disease interaction still developed from *Xoo* strains carrying respective TALE infecting rice lines with mutations in the corresponding EBEs of *OsSWEET* promoter. This possibly could be explained by the existence of novel effectors targeting other susceptibility genes or the capability of one effector to induce several susceptibility genes (Zhou et al. 2015; Xu et al. 2019). Recently, a diagnostic kit for blight resistance was made available which contains double- and triple-knock-out mutant lines to determine which *SWEET* gene is the target of a particular *Xoo* strain (Eom et al. 2019). These rice lines with multiple disruptions in different *S* genes as a result of genome editing approaches are potential genetic resources that will combat bacterial blight diseases.

Rice blast is a fungal disease caused by *Magnaporthe oryzae*. Ethylene responsive factors (ERFs) participate in resistant disease response against *M. oryzae* (Cao et al. 2006). ERFs are members of the AP2/ERF (APETALA2/Ethylene response element-binding factors) gene superfamily, known to play important roles in multiple abiotic and biotic rice stress responses (Abiri et al. 2017). *OsERF922* was reported as a negative regulator of blast resistance and is upregulated by both avirulent and virulent strains of *M. oryzae* (Liu et al. 2012). RNAi knockdown of *OsERF922* conferred resistance to blast disease (Liu et al. 2012). Mutant rice lines characterized by frameshift mutations in the coding region of *OsERF922* with enhanced resistance to *M. oryzae* were also developed using CRISPR/Cas9 (Wang et al. 2016). In the same study, mutations are shown to be transmitted to T1 and T2 generations with no significant changes in agronomic traits which demonstrate that this strategy can be used to produce blast-resistant rice lines.

Rice tungro disease (RTD) is a devastating rice viral disease caused by a mixed infection of *Rice tungro bacilliform virus* (RTBV) and *Rice tungro spherical virus* (RTSV). RTD accounts for 5% to 10% annual losses of rice yield in South and Southeast Asia (Dai and Beachy 2009). Resistance to rice tungro viruses is a rare trait among rice germplasm sources but identification of tungro virus resistance genes is significant in developing durable resistance to RTD (Azzam and Chancellor 2002; Dai and Beachy 2009). RTSV resistance is controlled by a single recessive gene encoding putative translation initiator factor 4G (*eIF4G*) (Lee et al. 2010). SNPs in codons for Y^1059^V^1060^V^1061^ of *eIF4G* were strongly associated with reactions to RTSV among rice genotypes. Macovei et al. (2018) used CRISPR/Cas9 targeted mutagenesis to induce mutations immediately upstream of the YVV residues in *eIF4G* of IR64, an indica cultivar susceptible to RTSV. The mutation was inherited in the next generation. The developed mutant lines showing resistance to RTSV could be used as source material for RTD resistance.

#### Wheat

A wheat line with TALEN-induced mutations in all three homeologs of *MLO1* exhibited complete resistance against *Blumeria graminis*, the causal agent of powdery mildew in wheat (Wang et al. 2014). This line was recently phenotyped in detail and compared to TILLING mutants providing partial resistance upon pathogen attack (Gruner et al. 2020). The authors report a correlation between the strength of powdery mildew resistance and susceptibility to the hemibiotrophic pathogen *Magnaporthe oryzae pv.* Triticum (MoT) causal of wheat blast disease, while the outcomes upon infection with *Zymoseptoria tritici* causing Septoria leaf blotch remained unchanged. Furthermore, it was found that edited plants do not show unwanted pleiotropic phenotypes with respect to callose deposition in leaves or early leaf senescence, as was detected in barley mlo mutants.

Enhanced disease resistance locus 1 negatively regulates powdery mildew resistance in Arabidopsis, and in order to test its function in wheat the three homeologs were targeted via Cas9 (Zhang et al. 2017). Using one sgRNA targeting all three homeologs, three plants with simultaneously edited alleles could be identified in T0, and homozygous mutant plants were identified in T1 generation. These plants exhibited reduced sensitivity to *Blumeria graminis* infection and reduced cell death. Although edr1 is not classified as a major disease resistance locus, its homozygous mutant status may contribute as breeding germplasm, considering that in Arabidopsis the edr1 mutant does not show constitutively activated defense responses and first phenotypic analyses of mutants in barley show otherwise non-compromised plant growth parameters (Zhang et al. 2017).

*FXL1*, a transcription factor used by *Fusarium* to repress defense responses in Arabidopsis upon its infection, is present in two copies on each of the wheat subgenomes (Brauer et al. 2020). The authors first showed that NFXL1 downregulation via RNAi in barley also confers partial resistance upon infection as had been shown in Arabidopsis. A sgRNA pair targeting all six loci simultaneously was then used to generate individuals with edits in all six homeologs in the T1 generation (one homeolog remained in heterozygous state). These showed increased Fusarium head blight resistance, similar to the RNAi line.

Kumar and colleagues revisited the findings that knocking out MORC genes in Arabidopsis has a negative effect on biotic stress resistance, in contrast to RNAi lines in barley (Kumar et al. 2018). They could confirm in detached leaf assays that knocking out *MORC1* leads to enhanced resistance against *Blumeria graminis* and *Fusarium graminearum*. Barley mutants with knocked out *hvmorc1* further showed de-repression of transposable elements which could not be seen in the RNAi lines but was observed in the Arabidopsis mutants, demonstrating that different strategies to create mutants may result in distinct outcomes. Another study addressing pest resistance showed that combined knock-out of two of three aphid-induced beta-1,3-glucanase genes led to increased callose formation in leaves, however the effect did not lead to enhanced aphid resistance (Kim et al. 2020).

The *eIF4E* (eukaryotic translation initiation factor 4E) gene and its isoforms are the most widely exploited recessive virus resistance genes. Recessive resistance is usually based on the loss or mutation of an essential host factor required by the virus, and the recessive alleles often encode proteins with abolished ability to interact with virulence factors (Dong and Ronald 2019). Editing of the eIF4E locus in GP and elite cultivars used in Kazakhstan has been reported (Kershanskaya et al. 2019).

### Herbicide tolerance

#### Maize

Prime editing was successfully applied to generate W542L and S621I double mutations in the genes encoding *ALS* genes in maize to develop herbicide resistance maize (Jiang et al. 2020). It was noted that prime-editing efficiency was improved by enhancing pegRNA expression with higher prime-editing efficiency compared to rice. Two pegRNA variants for creating W542L and S621I double mutations were compared in *ZmALS1* and *ZmALS2*. To reduce the number of pegRNA scaffold-derived byproducts, it was proposed that one to three nucleotides (C, GC, or TGC) of the pegRNA scaffold adjoining the RT template to be used as termination signals in genomic DNA for RT templates.

#### Rice

Butt et al. (2020) reported 0.26 to 2% efficiency of prime editing in the rice gene *ACETOLACTATE SYNTHASE* (*OsALS*) involved in herbicide tolerance. Sanger sequencing confirmed the editing according to the 15 bp RT template with two nucleotide substitutions, G-to-T substitution (W548L) and G-to-A substitution (silent mutation and destroys PAM site). Another mutation that is not based on the template showing A-to-G substitution was also identified and possibly originated from the scaffold RNA containing a ‘G’ next to the RT template. Nucleotide substitution of the rice endogenous acetolactate synthase (*ALS*) with a frequency of 14.3% (Lin et al. 2020b) up to 26% (Xu et al. 2020) were successfully targeted leading to the development of herbicide-tolerant rice.

### Abiotic stress tolerance

Drought stress is a significant problem in the rice-growing regions with inadequate irrigation facilities (Kamoshita et al. 2008). Mild and severe drought stresses during the reproductive stage in rice can cause 28% and 70% yield losses, respectively (Babu et al. 2003; Dixit et al. 2014). While salinity tolerance is important for low coastal regions and semi-arid inland saline areas of rice cultivations.

In a recent study, CRISPR/Cas9 targeted mutagenesis of the zinc finger transcription factor *OsDST* (*DROUGHT AND SALT TOLERANCE*) was used to develop mutant lines of indica mega rice cv. MTU1010 with tolerance to both drought and salinity stress (Santosh Kumar et al. 2020). Under drought conditions, *OsDST* mutant plants produced broader leaves with a lower stomatal density, thus showing improved water retention under dehydration stress (Santosh Kumar et al. 2020). The reduction of stomatal density in rice plants increases its ability to conserve water, as observed in the IR64 mutant lines overexpressing the rice epidermal patterning factor *OsEPF1* (*EPIDERMAL PATTERNING FACTOR*)(Caine et al. 2019). Another gene involved in the regulation of leaf stomatal density is *OsEPFL9* (*EPF-LIKE 9*). Successful knock-out of *OsEPFL9* using CRISPR/Cas9 and CRISPR/Cpf1 systems targeting the Exon 1 of the gene significantly reduced the stomatal count by more than an eightfold reduction in the mutant lines (Yin et al. 2017, 2019).

Leaf rolling plays an important role during drought stress in rice because it reduces water loss and decreases stomatal conductance. Targeted mutagenesis of *SEMI-ROLLED LEAF1,2* (*SRL1* and *SRL2*) genes were performed on three restorer rice lines (GXU16, GXU20, and GXU28) using a CRISPR/Cas9 vector targeting exons of *SRL1* and *SLR2* genes. Homozygous and heterozygous mutants were identified in T_0_ generation. T_2_ homozygous lines showed a rolled leaf phenotype until maturity. The mutant plants showed a lower transpiration rate and lower stomatal conductance than WT. The grain filling percentage under severe drought conditions was 27.5% and 2.5% in mutants and WT plants, respectively. The mutant plants showed a higher survival rate, abscisic acid (ABA) content, and antioxidant enzymes than WT plants under drought stress (Liao et al. 2019).

The CRISPR/Cas9 technique has been widely used to create knock-out or knock-down mutant lines to validate gene function. One example is the knock-out of *OsNAC14,* an important abiotic stress-responsive transcription factor expressed at the meiosis stage and induced by abiotic stresses including drought. It triggers subtle plant adaptation mechanisms to combat drought stress by reprogramming the transcriptional network. The CRISPR/Cas9 technique was used to validate the role of *OsNAC14* as a drought transcription factor (Shim et al. 2018). In contrast, to obtain drought tolerance in the vegetative stage of rice growth, increased expression of this gene is required, and it was demonstrated by overexpression of the *OsNAC14*. Editing the gene regulatory elements could potentially be used to increase the expression of drought transcription factors.

Respiration and photosynthesis in rice are affected by salinity; therefore, saline soil can cause major damage for rice plants throughout its life cycle. Mutants with targeted mutagenesis of *OsRR22* genes exhibited more salinity tolerance than WTs at the seedling stage of homozygous plants. There was no pleiotropic effect observed in the mutant populations (Zhang et al. 2019c).

Proline-rich proteins play an important role in osmotic stress tolerance as well as cold tolerance. Knocking out of the *OsPRP1* gene resulted in an increase in cold sensitivity and also demonstrated a low survival rate and reduced root biomass in the mutants (Nawaz et al. 2019). Accumulation of less antioxidant enzyme activity and lower level of abscisic acid (ABA), proline and ascorbic acid signifies the cruciality of *OsPRP1* gene in stress conditions. In the *OsPRP1* mutants, the expressions of anti-oxidant encoding genes were significantly down-regulated while there was an increase in activity of superoxide dismutase (SOD), peroxidase (POD), and catalase (CAT) under cold stress as compared to WT. Modulation of anti-oxidants and maintenance of signaling pathways crosstalk suggested that *OsPRP1* gene can be utilized for the improvement of cold tolerance in rice (Nawaz et al. 2019).

### Nutritional and quality traits

#### Maize

The *SHRUNKEN2* (*SH2*) and *WAXY* (*WX*) loci control the seed phenotype, with *sh2* mutations producing sweeter kernels due to the accumulation of sugar rather than starch and *wx* mutations altering the amylose/amylopectin ratio. Dong et al. (2019) transformed maize with a dual gRNA construct targeting *SH2* and *WX* simultaneously, recovering plants with either a single or double mutations producing sweet (*sh2*), waxy (*wx*) or sweet and waxy seeds (*sh2 wx*). Gao et al. (2020a) targeted the *WX* gene of an elite inbred maize line and recovered plants with 4-bp and 6-bp deletions at the target locus and no-off target mutations. Qi et al. (2020) also used CRISPR/Cas9 to mutate the *WX* locus in the ZC01-DTM*wx* background. The hybrid genome background of the resulting lines (determined using genome-wide SNP data) was up to 98.19% for lines used as male parents and up to 86.78% for lines used as female parents. Hybrids of both parental lines were similar to WT lines in terms of agronomic performance.

The distribution of sugar and starch in maize can also be modulated by interfering with sugar transport, as recently shown by using CRISPR/Cas9 to mutate three paralogs of the maize SWEET13 gene family (*SWEET13a*, *SWEET13b* and *SWEET13c*) which encode strongly expressed sucrose transporters in the leaf vasculature (Bezrutczyk et al. 2018). The comprehensive analysis of single, double and triple mutants revealed significant functional redundancy, with the single and double mutants showing minimal growth defects but the triple mutant showing an extreme developmental phenotype, including severe stunting and narrower, chlorotic leaves. The triple mutant leaves accumulated fivefold more starch than WT leaves, primarily in mesophyll and bundle sheath cells, and also more sucrose (twice as much at the leaf base and threefold more at the tip). The mutant leaves also accumulated sevenfold more glucose at the tip but there was no significant difference at the leaf base. These results are consistent with symptoms expected for impaired phloem loading (Bezrutczyk et al. 2018).

#### Rice

Several studies have used the CRISPR/Cas9 system to inactivate genes involved in starch biosynthesis, often causing not only the direct and anticipated impact on starch and sugar levels (which are strongly interrelated) but also pleiotropic effects. For example, knocking out the *SBEIIb* gene (encoding starch branching enzyme IIb, which is required for amylopectin synthesis in the endosperm) produced opaque seeds with depleted starch reserves. The total starch content in the mutant line was reduced by 26% and the amylose content increased from 19.6 to 27.4% of total starch. The mutation also had a broad effect on general primary and secondary metabolism in the endosperm, causing the accumulation of multiple sugars, fatty acids, amino acids and phytosterols compared to WT azygous controls, as discussed in subsequent sections (Baysal et al. 2020).

In another study, endosperm-specific inactivation of the *APL2* gene (encoding the cytosolic AGPase large subunit) induced the ectopic expression of *APL2* and the corresponding small subunit gene (*APS2b*) in leaves, but nevertheless reduced total starch levels in the leaves by 85% and increased the soluble sugar content by 40% (Pérez et al. 2018). The same group later showed that knocking out the *Waxy/GBSSI* gene reduced the amylose content of the endosperm to 5% in homozygous seeds and 8–12% in heterozygous seeds while increasing the soluble sugar content by 57%, resulting in fully translucent seeds (Pérez et al. 2019). Similarly, inactivating *PGM* (encoding plastidial phosphoglucomutase) and *APL4* (encoding the plastidial AGPase large subunit) inhibited starch synthesis and increased sugar levels in the seeds, although the precise values were not reported, as well as triggering male sterility and the complete abolition of pollen development (Lee et al. 2016).

In addition to genes involved in starch biosynthesis, CRISPR/Cas9 has also been used to inactivate genes involved in the regulation of sucrose/starch ratios. For example, knocking out the *SPS1* gene encoding sucrose phosphate synthase (SPS) reduced total SPS activity in the leaves by 46% without changing the sugar and starch content (Hashida et al. 2016). However, the double knock-out of *OsSPS1* and *OsSPS11* reduced total SPS activity in the leaves by 84% and caused the accumulation of leaf starch, although there was no significant impact on plant growth (Hashida et al. 2016). The sucrose to starch ratio is also influenced by sucrose translocation from the maternal tissue to the embryo, which is regulated in part by the sucrose transporter SWEET11. Ma et al. (2017) knocked out the *SWEET11* gene, reducing the sucrose concentration by 40% in the mutant embryo sacs, resulting in defective grain filling and a 5% drop in the starch content of the mature caryopses. Deng et al. (2020) generated single and double knock-outs of the sugar metabolism vacuolar invertase genes *INV2* and *NV3*. The grain size of the *inv2* mutant was normal, but that of the *inv3* mutant and *inv2-inv3* double-knock-out mutant was smaller, reducing the grain weight by 33.5%. In all knock-out mutants, the sucrose level was higher, but the total hexose content was lower. Furthermore, the total starch content was similar in the WT plants and knock-out mutants, but the amylose content of the mutants was 3–6% lower (Deng et al. 2020).

CRISPR/Cas9 system was utilized by Zeng et al. (2020b) in the development of a strategy that would regulate gene expression of *Wx* gene controlling amylose synthesis at the transcriptional and post-transcriptional level by editing the promoter and 5’UTR intronic splicing site (5′UISS), respectively. Modifications were targeted to the three putative *cis-*regulatory elements (CREs) and the 5’UTR region present in the 2 kb region upstream of the gene. Base insertions, base deletions or fragment deletions removing putative CREs were identified from 23 homozygous mutant T2 lines which resulted in reduced *Wx* gene expression in some of the transgenic lines. Different splicing patterns and reduced mRNA levels were also observed in the 5’UISS mutants. Agronomic traits were not significantly different compared to the control except for TGW.

Knocking out the *SBEIIb* gene (Baysal et al. 2020) caused the accumulation of multiple fatty acids, including a 1.3-fold increase in stearic acid (C18:0), a > 1.5-fold increase in myristic acid (C14:0), palmitic acid (C16:0), linoleic acid (C18:2) and behenic acid (C22:0), and a 2.6-fold increase in arachidic acid (C20:0). Furthermore, pentadecanoic acid (C15:0) was not detected in WT endosperm but accumulated to a concentration of > 2 µg/g in the mutant line. The potential health benefits of oleic acid have made it an important target for metabolic engineering, and Abe et al. (2018) therefore used CRISPR/Cas9 to knock-out the *FAD2-1* gene encoding the enzyme fatty acid desaturase 2 (FAD2), which catalyzes the conversion of oleic acid (C18:1) to linoleic acid. Rice bran oil typically contains a mixture of palmitic acid, oleic acid and linoleic acid, reflecting the composition of the seeds. While the fad2-1 mutant allows the production of bran oil with double the normal oleic acid content and no detectable linoleic acid.

Phospholipids and *Myo*-inositol 1,2,3,4,5,6-hexa*kis*phosphate (InsP6) or phytic acid (PA) or phytate are important phosphorus (P)-containing compounds in rice grains. However, phytate is an anti-nutritional factor that reduces phosphorus availability in the diet and increases the excretion of phosphorus-rich waste into the environment. Low-phytate rice grains are therefore better for the diet and prevent phosphorus-induced environmental damage. Khan et al. (2019) used the CRISPR/Cas9 system to generate mutants of a phospholipase D gene (*OsPLDα1*) and analyzed the mutational effect on metabolites, including PA in rice grains. Metabolic profiling of two *ospldα1* mutants revealed depletion in the phosphatidic acid production and lower accumulation of cytidine diphosphate diacylglycerol and phosphatidylinositol. The mutants also showed significantly reduced PA content when compared to their WT parent, and the expression of the key genes involved in the phytic acid biosynthesis was altered in the mutants. These results demonstrate that *OsPLDα1* not only plays an important role in phospholipid metabolism but also is involved in PA biosynthesis, most probably through the lipid-dependent pathway, and thus revealed a potential new route to regulate phytic acid biosynthesis in rice (Khan et al. 2019). PA also reduces the bioavailability of essential micronutrient such as Fe^2+^ and Zn^2+^ in cereal grain. Using CRISPR/Cas9 method, Jiang et al. (2019) generated knock-out mutants of an *ITP5/6K* homolog, *ITP5/6K-6*, by targeted mutagenesis of the gene’s first exon using the: one (ositpk6_1) with a 6-bp in-frame deletion, and other three with frameshift mutations (ositpk6_2, _3, and _4). These mutations significantly lowered PA content in rice grains. However, *ITP5/6K-6* gene knock-out also results in impaired plant growth. Thus, the use of the CRISPR/Cas9 system on the *ITP5/6K-6* gene may be more useful if knockdown lines are generated instead of a total knock-out of *ITP5/6K-6* expression.

The manipulation of amino acid metabolism by genome editing in rice has mainly focused on pathways that play a key role in nitrogen metabolism and transport. Luo et al. (2018) used CRISPR/Cas9 to knock-out the *ASN1* gene encoding asparagine synthetase. This reduced the concentration of asparagine to ~ 50% of WT levels in the root and shoot but also reduced the concentrations of glutamate (to ~ 50% of WT levels in the shoot and ~ 20% in the root) and aspartate to ~ 80% of WT levels in the shoot and ~ 15% in the root). In contrast, the concentration of glutamine increased fourfold in the root and two-fold in the shoot. These changes were not caused by nitrogen absorption because the nitrogen influx rate per unit weight did not change between the WT and mutant plants. The mutants were also one-third shorter than WT plants and produced about half the normal number of tillers. Knocking out the *SBEIIb* gene (Baysal et al. 2020) caused the accumulation of several amino acids, including seven that were not detected in WT endosperm (alanine, aspartic acid, glycine, lysine, proline, serine, and valine). Alanine, aspartic acid, and proline all accumulated to > 100 µg/g.

Gamma-aminobutyric acid (GABA) is a non-protein amino acid synthesized from glutamic acid by the enzyme glutamate decarboxylase (GAD). This is another important target of metabolic engineering in rice because it plays a key role in plant stress responses, growth, and development, and as a nutritional component of the grain can also reduce the likelihood of hypertension and diabetes. GABA-fortified rice was generated by using CRISPR/Cas9 to knock-out the *GAD3* gene (Akama et al. 2020). The analysis of free amino acids in the seed revealed a seven-fold increase in GABA levels as well as small increases in the levels of serine and glutamate, and twofold–fivefold increases in the levels of alanine, aspartate, methionine, phenylalanine, leucine and isoleucine. In contrast, the levels of asparagine and tryptophan fell to 30% of the WT level. The mutant also showed a higher seed weight (12%) and seed protein content (20%) than WT plants (Akama et al. 2020).

Carotenoids play an important role in the human diet, including the pro-vitamin A activity mostly provided by β-carotene. Carotenoids do not accumulate naturally in rice endosperm because the *PSY1* gene, encoding the first committed enzyme in the carotenoid biosynthesis pathway (phytoene synthase), is not expressed in this tissue. In an attempt to increase β-carotene levels in rice endosperm without introducing a *PSY1* transgene, Yang et al. (2017) used the CRISPR/Cas9 system to knock-out five genes involved in carotenoid catabolism (*CYP97A4*, *DSM2*, *CCD4a*, *CCD4b*, and *CCD7*), hoping to take advantage of any residual phytoene synthase activity, but there was no significant change in carotenoid levels. A putative rice ortholog of the *Orange* (*Or*) gene was also edited using CRISPR/Cas9, resulting in orange callus tissue in which β-carotene accumulated to 2.7 μg/g fresh weight (Endo et al. 2019).

The predominant extractable flavonoids in rice include flavone C-glycosides and flavone *O*-conjugates. Flavone C-glycosides exist in the form of apigenin, luteolin, or chrysoeriol C-glycosides and serve as phytoalexins, allelochemicals, feeding deterrents, and insect antifertility agents. Tricin-derived metabolites are the most abundant flavone *O*-conjugates, also functioning as allelochemicals and insect deterrents. Lam et al. (2019) edited the *CYP75B3* and *CYP75B4* genes involved in the biosynthesis of soluble flavone C-glycosides and tricin-type metabolites and analyzed the extractable flavonoid profiles. They found that apigenin levels increased by 74% and tricin levels increased by 14% in the *cyp75b3* knock-out lines, whereas in the double knock-outs apigenin levels increased by more than 100-fold but tricin was completely absent (Lam et al. 2019). Furthermore, 2-acetyl-1-pyrroline (2AP) is a major fragrance compound among the 100 or more volatile compounds that contribute to the flavor of cooked fragrant rice. TALENs were used to knock-out the *BADH2* gene encoding betaine aldehyde dehydrogenase, enabling the accumulation of 2AP in non-fragrant rice varieties, increasing the concentration from zero to 0.75 mg/kg (Shan et al. 2015).

A 14-bp frame-shift deletion in the seventh exon of the *Rc* gene, hence creating a premature stop codon is responsible for the white grain phenotype in most cultivated rice varieties. Targeted deletion of the sequences flanking the 14-bp deletion site was performed on three elite white pericarp rice varieties, including Xiushui134 (*japonica* inbred), Shuhui143 (*indica* restorer line) and ZhiNongS (*indica* two-line sterile line) through co-transformation of *Agrobacterium* strains harboring different CRISPR/Cas9 vectors. Plants with mutations that revert the 14-bp deletion to a deletion in multiples of 3 bases (15-, 18-, 30-bases) showed a change in the coloration of the grains from white to red, indicating the restoration of *Rc* gene function. Seeds harvested from T1 in-frame *Rc* lines showed a fivefold increase in proanthocyanidin and a 17-fold increase in cyanidin. There are no significant differences in major agronomic and grain quality traits between the in-frame *Rc* mutants and their corresponding WTs (Zhu et al. 2019).

Flavanone 3′-hydroxylase (F3′H), dihydroflavonol 4-reductase (DFR) and leucoanthocyanidin dioxygenase (*OsLDOX*) are among the enzymes involved in the biosynthesis pathway of anthocyanin. Targeted mutagenesis of *OsF3′H*, *OsDFR* and *OsLDOX* genes using three CRISPR/Cas9 vectors separately was performed on black rice cultivars, Heugseonchal and Sinmyungheugchal. The mutant lines showed changes in the seed color and 91–94% reduction in total anthocyanin content. Homozygous mutant lines lacking the T-DNA were identified in the T1 generation (Jung et al. 2019b).

The lignin composition of rice plants is important because it contributes to structural rigidity and could also allow the use of bagasse in the pulp and paper industry or for energy recovery. The structure of lignin reflects the ratio of syringyl and guaiacyl units, which is controlled by the enzyme coniferaldehyde 5-hydroxylase (CAld5H1). The CRISPR/Cas9 system was used to knock-out the *CAld5H1* gene, resulting in the accumulation of more guaiacyl units, increasing from 44 to 65% in the culm and from 79 to 96% in the leaf (Takeda et al. 2019). There were also increases in the levels of arabinan (28%), xylan (56%), and galactan (18%) relative to WT plants. Secondary cell walls of cellulose, hemicelluloses, and lignin are major components of rice biomass residues. The knock-out of *SND2*, encoding a transcription factor that regulates secondary cell wall development, reduced the cellulose content from 340 to 280 mg/g and downregulated the expression other genes related to secondary cell walls including *MYB86L*, *MYB61L* and *MYB58/63* (Ye et al. 2018).

Genome editing to develop low cadmium (Cd) rice using CRISPR/Cas9 technology was demonstrated by knocking out the metal transporter gene *OsNramp5* in *indica* rice. In hydroponics, Cd concentrations in shoots and roots of *OsNramp5* mutants were decreased. Knocked-out *OsNramp5 mutant* grains were consistently less than 0.05 mg kg^−1^, in contrast to high Cd concentrations from 0.33 to 2.90 mg kg^−1^ in grains of the WT under Cd contaminated field trials (Tang et al. 2017). The *OsNramp5* was also targeted in the *japonica* rice. However, in this study, the plant height was reduced in the generated mutants. The mutants also had lower seed setting rate and total seed number per panicle than WT. The grain yield in the mutants was also reduced to 76–85% those in WT. The Cd content in the grains also showed a marked reduction in the mutants compared to WT plants (Yang et al. 2019). Generation of mutants of *OsLCT1* and *OsNramp5* genes showed that the *OsNramp5* mutants have a higher level of Cd accumulation in the grains while the OsLCT1 mutants are safe for human consumption, accumulating less Cd in the grains (Songmei et al. 2019).

#### Wheat

The reduction or elimination of food allergens is another widespread goal of genome editing approaches, and the editing of α-amylase/trypsin inhibitor genes in durum wheat represents a recent example (Camerlengo et al. 2020). With respect to generally enhancing seed protein content, targeting *TaGW2* in the B1 and D1 genome led to an increase in grain protein content which had a positive effect on flour protein content and gluten strength, two important quality parameters (Zhang et al. 2018).

#### Barley

A frequently pursued breeding goal in cereal crops addresses phytate, an anti-nutrient. Non-ruminants depend on phytases for utilizing nutrients sequestered in phytate, such as phosphorus, iron, and zinc. Triticeae, in contrast to non-Triticeae cereals, show mature grain phytase activity (MGPA) while the latter rely on de novo synthesis during germination. Editing using TALEN and Cas9 validated *HvPAPhy-a* as a major MPGA contributor in barley seeds. Promoter editing led to alleles with mutations downstream of known transcription factor binding motifs and revealed promoter regions accounting for even higher transcriptional activity than the known motifs (Wendt et al. 2013; Holme et al. 2017). These findings may be used to breed for improved phytase activity. Alternative strategies rely on lowering phytate synthesis in seeds, for example by targeting a member of the inositol triphosphate 5/6 kinases (IPTK) comprising six genes in barley (Vlčko and Ohnoutková 2020). Inorganic phosphate content was measured in mature grains of edited *iptk1* mutants having either a 1 bp insertion or deletion or being bi-allelic for the mutations. However, a large variation in inorganic phosphate content was detected in the mutant plants, including individuals with the same allelic state.

D-hordein is highly homologous to the high molecular weight (HWM) glutenin subunits of wheat and its presence is thought to be negatively correlated with malting quality. Two studies targeted the corresponding genomic locus in barley and managed to isolate individuals devoid of D-hordein as evidenced by SDS-PAGE analysis (Li et al. 2020a; Yang et al. 2020). Another trait that is favored for brewing is low (1,3;1,4)-β-glucan content. To investigate enzymes contributing to high grain β-glucan content, two known and two putative (1,3;1,4)-β-glucan synthases were knocked out independently (Garcia-Gimenez et al. 2020). The authors could confirm the major role of HvCslF6 in grain β-glucan production. However, the edited plants also had a decreased TGW, altered grain morphology and a lower germination rate. In order to engineer well performing plants suitable for brewing, it was therefore suggested to target the promoter region instead or to alter specific sites in the HvCslF6 gene to induce more subtle changes with reduced pleiotropic effects.

With respect to starch accumulation in barley grain, CRISPR/Cas9-based editing was used to characterize the function of Protein Targeting to Starch 1 (PTST1) (Zhong et al. 2019a). HvPTST1 is localized around starch granules in barley endosperm and interacts with Granule Bound Starch Synthase I (GBSSI). Its overexpression leads to an increase in amylose content. PTST1 was found to be essential for grain starch accumulation as *ptst1* mutant grains formed wet endosperm devoid of starch and consequently grains were not able to germinate.

## Application of genome editing in hybrid breeding

Male sterile lines are valuable resources for maize hybrid seed production, and genes that control male fertility were therefore among the early targets of genome editing during the proof-of-principle studies described at the beginning of this section, including *MS26* (Djukanovic et al. 2013; Svitashev et al. 2015, 2016) and *MS45* (Svitashev et al. 2015, 2016). More recently, CRISPR/Cas9 has been used to mutate other male fertility genes, including *MS8* (Chen et al. 2018), *MS33* (Xie et al. 2018), and *TMS5* (Li et al. 2017b), the latter producing thermosensitive *tms5* male-sterile mutants. Young et al. (2019) developed a comprehensive in silico and experimental strategy for the precise targeting of *MS26* and *MS45* involving the prediction of target specificity using Cas-OFFinder, the biochemical capture and identification of genomic sequences susceptible to Cas9-induced DSBs using CLEAVE-Seq, and off-target site validation in plants. The authors designed gRNAs based on the outcome of this analysis and concluded that off-target editing is negligible when gRNAs are carefully designed, and indeed occurs at a much lower frequency than naturally occurring diversity in plants.

Doubled haploid lines are integral to many commercial maize breeding programs because they accelerate the development of pure-breeding parental lines that are used to produce hybrid seed (Chaikam et al. 2019). The typical approach involves the induction of maternal haploids by a male haploid-inducer genotype (Stock6 or a derivative such as CAUS) followed by chromosome doubling and selfing. Stock6 features two QTLs that promote a high haploid induction rate (*qhir1* and *qhir8*). Genome editing provides a shortcut to the development of male haploid-inducer genotypes by allowing the genes at these QTLs to be targeted directly, or by targeting other genes involved in chromosome pairing/separation and cell division. For example, TALENs were used in the non-inducer NP2222 background to create loss-of-function alleles in the *MATRILINEAL* (*MTL*) gene, encoding a pollen-specific phospholipase. This is a major gene contributing to the effect of *qhir1*. The resulting frameshift mutations led to a 6.7% haploid induction rate that could significantly accelerate the breeding of maize haploid lines (Kelliher et al. 2017). Similarly, Zhong et al. (2019b) identified the *DMP* gene by map-based cloning in *qhir8* and used the CRISPR/Cas9 system to introduce mutations affecting the haploid induction rate. Although a knock-out mutation only marginally increased haploid induction, a single-nucleotide change increased the rate by up to threefold alone, and by up to sixfold when combined with the *mtl* mutation, indicating additive or even synergistic effects between the loci. The efficiency of haploid induction might be enhanced further by the direct functional analysis of genes involved in chromosome pairing and separation, as recently shown by the generation of CRISPR/Cas9 mutations in the *STRUCTURAL MAINTENANCE OF CHROMOSOME3* (*SMC3*) gene revealing a previously unknown role in meiotic centromere pairing in addition to sister chromatid cohesion (Zhang et al. 2020a).

The applications of genome editing in maize breeding programs have been simplified by the development of the haploid inducer mediated genome editing system (IMGE). This allows the introduction of mutations in the maternal genome from a paternal CRISPR/Cas9 cassette which is subsequently eliminated naturally (Wang et al. 2019d). In traditional breeding programs, traits must be introgressed from varieties such as B104 (which are easy to transform) into elite lines by repeated backcrossing over at least six generations. Using the IMGE system, the same can be achieved in two generations if a haploid-inducer line carrying CRISPR/Cas9 is used to pollinate the non-inducer line. Genome editing occurs in both parental genomes but the paternal chromosomes (carrying the CRISPR/Cas9 transgene) are fragmented and eliminated after fertilization, allowing the rapid formation of double haploids carrying the mutation. Wang et al. (2019d) confirmed the IMGE principle by targeting the genes *LG1* and *UB2* in double haploid lines.

## Application of genome editing in precise transgenesis

### Precise targeted addition of multiple transgenes

Targeted insertion of transgenes at pre-determined plant genomic safe harbors provides a desirable alternative to insertions at random sites achieved through conventional methods. The targeted insertion of a 5.2 kb carotenoid biosynthesis cassette which consists of both transit peptide of pea RUBISCO small sub unit (*SSU)- carotene desaturase (CrtI)* encoding a multifunctional enzyme covering several steps in the endogenous pathway leading up to β-carotene and *Zea mays phytoene synthase* (*ZmPsy1)* encoding phytoene synthase, both driven by the endosperm-specific glutelin promoter, at two genomic safe harbors in rice was achieved by the use of an optimized CRISPR/Cas9-based method (Dong et al. 2020). The obtained marker-free rice plants have high carotenoid content in the seeds (7.90 μg g^−1^) and no detectable penalty in morphology or yield. Whole-genome sequencing reveals the absence of off-target mutations by Cas9 in the engineered plants. These results demonstrated targeted gene insertion of marker-free DNA in rice using CRISPR/Cas9 genome editing and offered a promising strategy for genetic improvement of rice and other crops (Dong et al. 2020).

### Transgene stacking

As discussed above, genome editing can be used to knock-in cassettes by HR at a given target site, such as the I-SceI site used in the original study (D’Halluin et al. 2008). However, if the knock-in cassette also carries an appropriate landing pad, then the process can be repeated over and over to introduce additional transgenes at the same site. This concept was developed by Dow AgroSciences using ZFNs, which is important because ZFN constructs with different targeting preferences can be used in each round to avoid the possibility of removing the previously integrated transgenes (Ainley et al. 2013). Transgene stacking was demonstrated by sequentially introducing two herbicide tolerance genes: *pat* encoding phosphinothricin acetyltransferase (tolerance to IgniteR) and *aad1* encoding aryloxyalkanoate dioxygenase (tolerance to AssureR II). One drawback of this method is that a different selectable marker is required to confirm each transgene knock-in event. The demonstration of stacking with herbicide tolerance genes sidesteps this issue because these genes also act as selectable markers, but for other traits, it would be necessary to introduce a different selectable marker along with every transgene. Kumar et al. (2015) designed a strategy to overcome this by incorporating the landing pad within an intron immediately downstream of the promoter driving the selectable marker gene, allowing the selectable marker and ZFN recognition site to be deleted after the first round of targeting by a donor carrying a new recognition site (also making it suitable for another round of targeting). The process can therefore be repeated over multiple cycles in order to stack several different transgenes without a commensurate number of different selectable markers.

The most recent development in this field is the complex trait locus (CTL) approach, which involves the engineering of maize plants with a local cluster of landing pads (Gao et al. 2020c). This is an extension of the safe harbor concept, which involves the integration of a single landing pad within an active genomic region. A CTL is created by generating multiple lines carrying landing pads at different sites within a small and well-characterized region in the genome. The landing pads are created by genome editing using the CRISPR/Cas9 system, which can also be used to knock in transgenes at these sites. The unique feature of the CTL approach is that the landing pads and their transgene passengers are then stacked by conventional genetic crossing. Because all the landing pads are present in the same chromosomal region, the transgenes can be introgressed as a single locus in breeding programs. However, the distance between the landing pads is optimized to allow not only group introgression but also the removal of individual traits by segregation if necessary.

## Multiplexed genome editing

Maize

Multiplex genome editing is widely practiced in cereals, and maize was used as a model during some of the first proof-of-principle studies in cereals, as discussed above (Svitashev et al. 2015). The tRNA-based processing of multiple gRNAs was pioneered in rice (Xie et al. 2015) but also demonstrated soon afterwards in maize (Qi et al. 2016). The results demonstrated that targeting one gene with two gRNAS using multiple tRNA-gRNA units increased the efficiency of gene knock-out in maize compared to simplex editing systems.

A binary vector system (ISU Maize CRISPR) was designed to target two maize gene families: Argonaute 18 (*AGOA* and *AGO18B*) and dihydroflavonol 4-reductase (*ANTHOCYANINLESS1* and *ANTHOCYANINLESS4*). For each gene family, with members on two different chromosomes, two gRNAs were designed to target two sites within each allele. T0 transgenic events carrying one or two mutations at one locus and various combinations of allelic mutations at two loci were recovered with a frequency of > 70% in both Hi-II and B104 backgrounds (Char et al. 2017).

In a more ambitious study, 20 genes representing several families as well as individual genes were targeted using various combinations of 28 gRNAs expressed from 12 plasmids, resulting in the recovery of 93 mutant alleles affecting 18 of the genes, and a 19% frequency of biallelic mutations (Doll et al. 2019). The most common mutations were small indels (< 10 bp) but the use of multiple gRNAs also resulted in the recovery of some larger deletions. There was a high frequency of double and triple mutants and no off-target mutations were detected, although only three potential off-target sites were checked.

Rice

Multiple gene editing in rice has also led to an improvement of yield attributing characters. Many QTLs in rice have been shown to affect grain number and morphology, the first of which were *Gn1a* (*GRAIN NUMBER 1a*) affecting grain number (Ashikari et al. 2005) and *GS3* (*GRAIN SIZE 3*) affecting grain size (Fan et al. 2006). As mentioned above, CRISPR/Cas9 system has been used to target these loci directly as a strategy to improve yield traits. The architecture of the variants in *GS3*, *Gn1a*, *DEP1 (DENSE AND ERECT PANICLE)* and *IPA1* (*IDEAL PLANT ARCHITECTURE 1*) genes of cultivar Zhonghua 11 produced larger, long-awn grains, more grains, and a dwarf stature with denser erect panicles (Li et al. 2016). Furthermore, the tiller number was affected in the *ipa1* mutants, but the number could be more or less than normal depending on the changes induced in the region targeted by the regulatory microRNA *miR156* (Li et al. 2016). The similarity of the mutant phenotypes to previous reports indicated that the four edited genes are suitable targets in many different genetic backgrounds (Li et al. 2016). The use of CRISPR/Cas9 for the multiplex editing of *GW2* (*GRAIN WIDTH 2*), *GW5*, *GW6* and *GS3*, encoding negative regulators of grain weight, resulted in a significant improvement in grain weight and size (Xu et al. 2016). The *gw5tgw6* double mutant showed increases of 11.69%, 8.47% and 12.68% in grain length, grain width and TGW, respectively, whereas the four *gw2gw5tgw6* triple mutants showed increases of 20–30% in the same traits, indicating that mutations in these QTLs had the anticipated additive effects (Xu et al. 2016). Grain size is also influenced by the gene *RGG2*, which encodes a type B Gγ subunit that negatively regulates plant growth and organ size in rice. The knock-out of this gene in a Zhenshan 97 (ZS97) background enhanced growth, including longer internodes, a 12% increase in TGW and a 16% increase in yield (Miao et al. 2019). In another study, targeted mutagenesis of *Gn1a* and *OsDEP1* genes resulted in mutants being superior in yield than the WT. Specifically, one mutant allele of the *Gn1a* gene and three mutant alleles of the *OsDEP1* gene conferred a higher yield than the WT. *Gn1a* and *OsDEP1* mutants showed an increase in the number of panicles per plant, which ultimately led to a higher number of grains per plant, with approximately 13–24.7% higher yield. Though further studies have been advised to observe succeeding generations (Huang et al. 2018). In an attempt to edit major QTLs, *GS3* and *Gn1a* resulted in the generation of *GS3* and *GS3-Gn1a* double mutants with no *Gn1a* mutants. Three genotypes (GS3-N9108, GS3-Z22, GS3-Gn1a-Z22) had higher grain yields (3–7%) than the WT (Shen et al. 2018).

Another study found that multiplex genome editing of *OsGS3, Gn1a,* and *OsGW2,* which generated single, double and triple mutant populations of three *japonica* rice elite rice varieties (J809, L237, CNXJ), resulted in yield improvement. Triple mutants resulted in a higher number of flowers per panicle per plant than the WT. The grain length in the triple mutants also increased by about 2 mm (length) and 1.5 mm (width). Triple mutants of J809 and L237 ultimately resulted in an increase in yield per panicle by 68% and 30% respectively (Zhou et al. 2019).

An essential phytohormone for growth and stress response is ABA. Studies have shown that *PYL* (Pyrabactin resistance 1-like genes), a sub-family of abscisic acid receptor genes, can lead to promoted growth and productivity in rice. Multigene knock-outs diverged in two classes, Group I (*PYL1*–*PYL6*, *PYL12*) and Group II (*PYL7*–*PYL11*, *PYL13*). Group I genes turned out to be more important for stomatal development, seed dormancy and plant growth regulation than Group II. The *PYL1* and *PYL12* exhibited significant defects in seed dormancy. In natural field conditions, among all the generated mutants, only the Group I mutants*, PYL1*, *PYL4*, *PYL6*, exhibited improved grain productivity (approximately 25%) than the WT (Miao et al. 2018).

*FWL* (FW 2.2-like) genes, genes encoding for cytosine-rich proteins, have vital roles in cell division, organ size control, rhizobium infection response and homeostasis of metal-ions in plants. A recent study, which performed targeted mutagenesis of rice FW 2.2-like gene, highlighted the regulatory role the *OsFWL4* gene plays for tiller numbers in *japonica* rice plants. The study found that *OsFWL4* is a negative regulator of tiller number and plant yield in rice, while the *OsFWL1* gene plays a role in modulating rice grain length. The number of tillers per plant and flag leaf width in *OsFWL4a* and *OsFWL4b* mutants was correspondingly 45.9% and 41.1% greater and was 7.7% and 6.3% higher than that of WT. Plant height, leaf size, and grain yield per plant for the mutants were not considerably different from the WT (Gao et al. 2020b).

A combination of abiotic tolerance as well as yield enhancement was performed by another current multiplex genome editing, performed on *Nipponbare* rice. *OsPIN5b* (a panicle length gene), *GS3* (a grain size gene), and *OsMYB30* (a cold tolerance gene) were targeted and the mutants exhibited increased enlarged grain size, panicle length, and increased cold tolerance respectively. The increase in panicle length was due to an increase in Auxin levels in the *OsPIN5b* mutants. The plant survival rates of *OsMYB30*-7 and *OsMYB30*-11 (66.7% and 70.8% respectively) were higher than the WT (41.7%). *OsPIN5b/GS3/OsMYB30-4* and *OsPIN5b/GS3/OsMYB30*-25 had survival rates that were higher (70.8% and 79.1%, respectively) than that of the WT (45.8%) (Zeng et al. 2020c).

Wheat

**Fig. 3:**
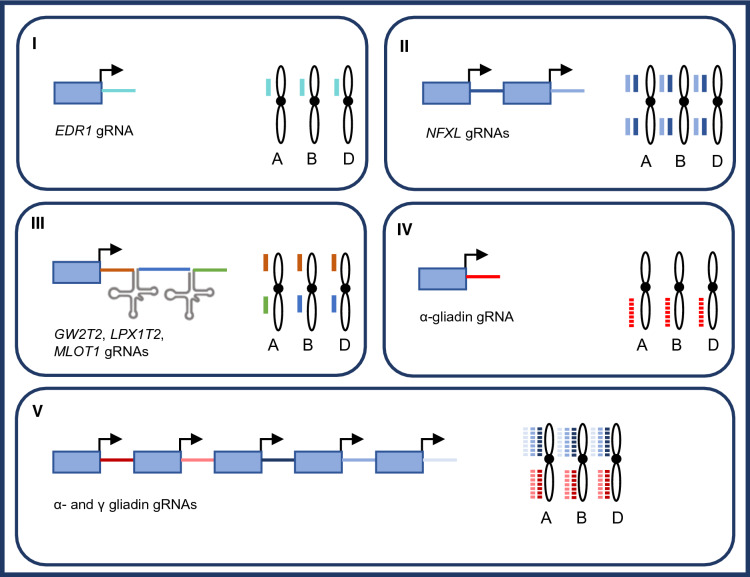
Examples of sgRNA programming for targeting multiple genomic sites simultaneously in hexaploid wheat. **I** One single guide RNA targeting all three homeologs (Zhang et al. 2017). **II** Multiplexing with two sgRNAs, each targeting two different sites in six homeologs (Brauer et al. 2020). **III** Multiplexing using a tRNA-gRNA polycistronic construct to target three, two and one homeologs of unrelated genes (Wang et al. 2018a, b). **IV** Using a single sgRNA to target multiple copies of alpha-gliadin gene copies (Sánchez-León et al. 2018). and **V** Multiplexing using five sgRNAs driven as separate cassettes and targeting alpha- and gamma-gliadin genes (Jouanin et al. 2019). **A**, **B**, **D** Label the sub genomes of bread wheat. Blue boxes with arrow: promoters driving sgRNA expression. sgRNAs are color-coded according to their ability to target the same genetic locus (across all three subgenomes, i.e. homeologs) or different genetic loci. The genomic locus targeted by sgRNAs is depicted by vertical bars or squares next to the subgenomes. gRNAs targeting the same locus but at different sites are depicted as parallel vertical bars with graded colours. For visualization purpose, the chromosome positions of the depicted loci are not drawn to match their relative actual locations

Due to the hexaploid nature of the bread wheat genome it is often necessary to target two or all three homeologs in order to achieve a desired phenotype. Since these in many cases share conserved sequences it is often possible to use one pair of TALEN (Wang et al. 2014) or one specific gRNA (Fig. 3i) to target all three loci simultaneously (Zhang et al. 2017). To elucidate NFXL1 activity in Fusarium resistance in wheat for example, three pairs of homeologs had to be targeted (Brauer et al. 2020). The authors decided to use two gRNAs, that had performed best in wheat protoplast assays and were complementary to two different regions present in all six NFLX1 loci, and expressed those, each separately driven by a wheat U6 promoter (Fig. 3ii). This strategy resulted in three edited plants in T1 (from two independent events) with editing in all six homeologs of *TaNFXL1* (biallelic/homozygous mutant for five of the six loci). Seeds of these plants were used for phenotyping. An alternative multiplexing approach, the polycistronic tRNA-gRNA system, was used in durum wheat for editing of two alpha-amylase/trypsin inhibitor genes, giving rise to seven gRNAs at once (Camerlengo et al. 2020). The same multiplexing system was used in a more experimental setup targeting *TaGW2*, *TaLpx-1* and *TaMLO*. Selective editing of all three, two, or only one of the three gene homoeologs, respectively, could be shown (Fig. 3iii) (Wang et al. 2018b). Out of 39 Cas9 positive plants one mutant homozygous for all three targeted loci of *TaGW2* and for the single targeted *MLO* locus on subgenome A could be identified using NGS amplicon sequencing, but there was only weak editing activity detected at the *TaLpx-1* locus in this individual. However, due to ongoing editing activity, termed “transgenerational activity”, a T2 individual with a fixed edited *TaLptx-1* allele at homeolog B could be identified, but editing activity at homeolog D remained low, highlighting the complexity of multiplexing in wheat and other polyploid species.

A particularly complicated challenge is the elimination of immunoreactive gluten components in wheat. Gliadin genes are encoded as multigene-families on all three subgenomes and alpha-gliadin alone is encoded by about 100 genes and pseudogenes (Sánchez-León et al. 2018). RNAi approaches have been used before to downregulate this multigene family. Two gRNAs each expressed from the wheat U6 promoter were programmed against conserved regions next to the immunodominant epitopes and transformed independently into two bread and a durum wheat cultivar (Fig. 3iv). NGS sequencing of amplicons covering the gRNA sites could identify 45 and 52 and 43 different, highly represented alpha-gliadin sequences in the cultivars, respectively. Sequencing of 17 T1 individuals revealed 35, 13 and 29 of those to be edited. The editing efficiency in T1 individuals varied between cultivars and gRNA used (highest up to 75% edited reads in one cultivar, others between 1,5%—14,7%). A second study aiming to establish low gluten wheat lines targeted alpha- and gamma-gliadin loci using a multiplexing approach involving five gRNAs, two targeting alpha- and three gamma-gliadin genes, in separate cassettes on a vector (Fig. 3v) (Jouanin et al. 2019). Finally, fragment deletions can be engineered also in wheat targeting a pair of sgRNAs to the same gene (Cui et al. 2019).

Barley

Multiplexing approaches in barley have been carried out to increase the chance for disrupting a given gene or for the generation of fragment deletions at a particular locus (Kapusi et al. 2017; Kapusi and Stöger 2018; Li et al. 2020a; Zeng et al. 2020a), or for the generation of double mutants at two loci (Gasparis et al. 2018; Kim et al. 2020). Different strategies were used to deliver the gRNAs: a polycistronic tRNA-sgRNA cassette (Gasparis et al. 2018; Zeng et al. 2020a), two conventional sgRNA cassettes on one vector (Kim et al. 2020; Li et al. 2020a) and mixing agrobacterium strains transformed with a vector containing each a single sgRNA cassette or, in the case of particle bombardment, co-bombardment of the two vectors (Kapusi et al. 2017; Kapusi and Stöger 2018). The probability of isolating individuals edited at two or more target sequences results from the combined efficiencies of the sgRNAs. Reported efficiencies for two gRNAs (number of double mutant T0 individuals per total T0 mutant individuals) are for example 4,7% (Kim et al. 2020) and 21% (Gasparis et al. 2018). Fragment deletions between sgRNAs placed near each other at a locus have also been achieved (90–139 bp in size, in 24% of mutant T0 individuals across several experiments) (Kapusi et al. 2017). An additional advantage of fragment deletions is the possibility of easy screening by PCR and agarose gel electrophoresis (Kapusi and Stöger 2018).

##  The role of the nuclease promoter 

The efficiency of genome editing is in part dependent on the promoter(s) used to drive the expression of the components, particularly in the case of the CRISPR/Cas9 system because protein and RNA components are required. In maize, the Cas9 protein or its equivalent is typically expressed under the control of a strong constitutive promoter from an endogenous protein-coding gene, usually the *UBIQUITIN1* (*UBI1*) promoter and first intron (Feng et al. 2018). The cauliflower mosaic virus 35S promoter has also been used (Feng et al. 2016), but the maize *UBI1* promoter is more efficient. Several different promoters have been used to drive the gRNA cassette, including the rice or wheat U3 snRNA promoters (Liang et al. 2014; Xing et al. 2014) and the rice or maize U6 snRNA promoters (Svitashev et al. 2015; Char et al. 2017; Li et al. 2017b). Where combinations have been compared, different sgRNA promoters combined with the *UBI1* promoter for Cas9 have resulted in significant variations in the efficiency of editing, suggesting that the screening of different promoter combinations would be beneficial if the efficiency of genome editing is low (Feng et al. 2018).

As an alternative to constitutive promoters, the nuclease component of the genome editing system can be expressed in a tissue-specific or inducible manner to restrict the genome modifications in time or space. An example of the former is the maize *DMC1* promoter which is active only in cells undergoing meiosis (Klimyuk and Jones 1997), therefore providing an inbuilt mechanism to avoid the generation of mosaics (Feng et al. 2018). This was tested against the *ZB7* gene, which generates an albino phenotype when mutated, resulting in a targeting efficiency of 100% including 66% biallelic mutations and no off-target mutations. An example of inducible genome editing in maize is the dexamethasone-inducible I-SceI described by Ayar et al. (2013). A more recent example is the *HSP26* promoter used to induce GT in maize by heat shock, including the introduction of DSBs and the release of a donor template to facilitate allele replacement for the repair of a selectable marker (Barone et al. 2020).

The soybean HSP17.5E promoter was used to drive the expression of Cas9 to induce targeted mutagenesis in rice by heat shock (Nandy et al. 2019). Targeted mutagenesis was suppressed in the regenerated plants but induced after heat shock treatments and the mutations were transmitted to the progeny at a high rate. When compared to the constitutive‐overexpression CRISPR/Cas9 lines, the heat shock-CRISPR/Cas9 lines showed lower rate of off-target mutations.

## Future perspectives and remaining challenges

Genome editing holds great promise for crop improvement. A significant advantage of CRISPR/Cas9 editing compared to earlier editing techniques is the possibility of obtaining multiple and precise modifications, and this technique is relatively more straightforward and economical. A range of traits has been edited in cereal crops using CRISPR/Cas9, ranging from traits with agronomic importance for the farmer to improved grain quality and nutritional benefits to the consumers. Continuous discovery of the new protein scissors with different protospacer adjacent motives such as CRISPR-Cas 12a in combination with Cas9 has increased the range of technical possibilities and positively contributed to editing different genes and regulatory elements more efficiently. De novo domestication of wild species through genome editing has been proposed as a new route to develop a new staple cereal that adapts well to the extreme environmental stresses, as shown recently in rice (Yu et al. 2021).

To date, the majority of products fall under the regulatory framework for SDN-1, which in many countries is considered *en-par* with those applying to conventional breeding products. Most of these products, particularly in rice, were developed through the error-prone NHEJ DSB repair mechanism. In contrast, the success of allelic replacement to accelerate trait introgression from a donor parent to popular recipient cultivars by HDR using a donor template (SDN-2) is still very limited; most of the successful studies were conducted for a proof of concept using a selective trait such as herbicide tolerance or a visible phenotype, due to its low frequency. Further innovation by overcoming the predominant NHEJ DNA repair pathway is required to increase HDR frequency.

An improved approach to creating desirable SNPs mimicking allele replacement by base editing or prime editing will be beneficial for its precision and reducing the potential of off-target editing. In addition, it does not involve a donor template that circumvents the limitation of particular countries' regulations to be exempted from genetic engineering's regulatory approval process. However, similar to SDN-2, these newly developed technology success stories are so far limited to the herbicide-resistant trait. The current prime editing system can only be used to modify a single gene. The large size of prime editing construct (~ 20 kb) may limit the efficiency of transformation into plant. When prime editing can be widely applied in multiple cereal crops efficiently, it will be a game-changer.

Facilitation of in-country product-based regulatory approval framework and cost of the authorization feasible for public sectors and its harmonization between multiple regions are the keys to future adoption of genome editing products. It will prevent possible trade disruption, allowing farmers and consumers to reap the benefits of gene editing in the future.

## Supplementary Information

Below is the link to the electronic supplementary material.

Supplementary file1 (DOCX 32 KB)

Supplementary file2 (DOCX 46 KB)

Supplementary file3 (DOCX 20 KB)
