# Supplementary material for: Genome editing in cereal crops: an overview
Source: Transgenic Res. 2021 Jul 14;30(4):461–98. doi: 10.1007/s11248-021-00259-6 (PMC8316241; doi:10.1007/s11248-021-00259-6)
Supplement: Supplementary file 1 — Supplementary file1 (DOCX 32 KB) [file 11248_2021_259_MOESM1_ESM.docx]

**Supplementary Table 1. Maize**

| **Method** | **Target** | **Knockout, insertion or replacement** | **Notes** | **Anticipated phenotype** | **Achieved phenotype** | **Pleiotropic effects (morphology)** | **Pleiotropic effects (molecular)** | **Reference(s)** |
| --- | --- | --- | --- | --- | --- | --- | --- | --- |
| CRISPR | *ARGOS8* | Insertion and replacement | Insertion of GOS2 promoter upstream of ARGOS8 gene, or replacement of native ARGOS8 promoter with GOS2 promoter | Increased grain yield due to reduced ethylene sensitivity | Increased grain yield in field trial | Not reported | Not reported | Shi et al 2017 |
| EMN (based on I-*Cre*I) | *LG1* upstream site (*lig34*) | Indels | Mostly short deletions (2-220 bp) centred around target site | Molecular test only | Molecular test only | Not reported | Not reported | Gao et al 2010 |
| CRISPR/Cas9 vs EMN | *LIG, ALS1, ALS2, MS26* and *MS45* | Indel (*LG1*), knockout (others) and replacement (*ALS2*) | Proof of concept and system comparison | Molecular test only | Molecular test only | Not reported | Not reported | Svitashev et al 2015 |
| CRISPR | *LG1* | Knockout | Desired target mutator (DTM) system, avoids linkage drag during maize breeding programs | Upright leaves | Upright leaves | Not reported | The DTM system induces mutations by delivering the CRISPR/Cas9 components in pollen | Li et al 2017a |
| CRISPR | *STIFF1* | Knockout | Multiplex editing with two gRNAs | Lodging resistance | Lodging resistance | Not reported | Not reported | Zhang et al 2020c |
| CRISPR | *PHYC1* and *PHYC2* | Knockout | Double knockout mutants | Early flowering time and stunting | Moderate early flowering under long-day condition but mild stunting only present in one line |  | ZCN8 was induced the mutants under LD conditions. The CONZ1 waveform in the mutant correlated well with that of ZCN8 under LD conditions (peak at midnight) | Li et al 2020b |
| CRISPR | *CCT9* | Knockout | Two 20-bp sequences in the first and second exons of *CCT9* were selected | Flowering delay | Null mutants flowered significantly earlier than wild-type | Not reported | Not reported | Huang et al 2017 |
| CRISPR | *XLG1, XLG3a* and *XLG3b* | Knockout | Multiplex editing with three gRNAs | Potential developmental defects | Single mutants showed no phenotype. Double mutants were slightly stunted. Triple mutants showed developmental arrest and died at the seedling stage | Not reported | Not reported | Wu et al 2018 |
| CRISPR | *GA20OX3* | Knockout | Multiplex editing with two gRNAs | Semidwarf | Semidwarf, reflecting decrease in cell numbers. GA12 and GA53 levels up, but most GA precursors depleted, as well as bioactive GA1 and GA4 | Semidwarf of mut1 and mut2 caused the decrease in cell numbers | Not reported | Zhang et al 2020b |
| TALEN | GLOSSY2 (GL2) | Knockout and precise insertion | Proof of concept | Glossy leaves | Glossy leaves | Not reported | Not reported | Char et al 2015 |
| CRISPR/Cas9 vs CRISPR/Cas12 | *GL2* | Knockout | Up to 100% of Cas9 mutations were indels whereas Cas12a favored larger deletions. Cas9 also more efficient for homozygous / biallelic mutations | Glossy leaves | Glossy leaves | Not reported | Not reported | Lee et al 2019b |
| CRISPR | *LG1, UB2* | Knockout | Haploid inducer mediated genome editing system | Upright leaves for LG1 | Upright leaves for LG1, UB2 data not provided | Not reported | Not reported | Wang et al 2019d |
| CRISPR | *BR2* | Knockout |  | Semidwarf | Semi-dwarf | Not reported | Not reported | Bage et al 2020 |
| CRISPR | *PT7* | Knockout |  | Function of PT7 was unknown | Defects in vegetative growth, lower dry weight, and shorter leaves | Not reported | Not reported | Wang et al 2020a |
| ZFN | *IPK1* | Marker gene insertion and simultaneous target gene knockout by insertional disruption | Simultaneous expression of ZFNs and delivery of a simple heterologous donor molecule leads to precise addition of an herbicide-tolerance gene at the target locus | Herbicide tolerance and lower inositol phosphokinase activity | Herbicide tolerance and lower phytate levels in seeds | Not reported | No off-target events | Shukla et al 2009 |
| MN I-SceI | Reactivation of a marker by knocking in a promoter at an I-SceI target | Promoter insertion | Target sequence insertion by homologous recombination | Herbicide tolerance | Herbicide tolerance | Not reported | Not reported | D’Halluin et al 2008 |
| ZFN | Targeted DNA integration for subsequent transgene stacking | Precise insertion | Landing pad with *pat*, gene stacking with *aad1* | Herbicide tolerance | Herbicide tolerance | Not reported | Not reported | Ainley et al 2013 |
| CRISPR | *SH2* and *WX* | Knockout | Multiplex editing with two gRNAs targeting different genes | Super-sweet and/or waxy seeds depending on combination of alleles at each locus | Super-sweet and/or waxy seeds depending on combination of alleles at each locus | Not reported | Not reported | Dong et al 2019 |
| CRISPR | *WX* | Knockout | Multiplex genome editing to generate deletions of 4–6 kb | Waxy seeds | Waxy seeds | Not reported | No pleiotropic effects reported, but mutations were induced in 12 elite maize inbreds | Gao et al 2020a |
| CRISPR | *WX* | Knockout | Desired target mutator (DTM) system, avoids linkage drag during maize breeding programs | Waxy seeds | Waxy seeds | Not reported | Not reported | Qi et al 2020 |
| CRISPR | *SWEET13a*-c | Knockout | Single, double and triple knockouts tested | Disruption of sugar/starch partitioning due to impaired phloem loading. | Triple mutants accumulated 5-fold more starch and up to ~4-fold more soluble sugars in the leaves, consistent with impaired phloem loading. | Single and double mutants showed minor growth defects. Triple mutants severely stunted with deformed and chlorotic leaves. | Not reported | Bezrutczyk et al 2018 |
| CRISPR | *bZiP22* | Knockout |  | Depletion of 27-kD γ-zein | Depletion of 27-kD γ-zein, slight increase in 50-kD γ-zein | Fewer protein bodies, and disruption of protein body morphology | Higher content of amylose, lysine, tryptophan  and methionine | Li et al 2018a |
| EMN (based on I-*Cre*I) | *MS26* | Knockout |  | Male sterility | Male sterility |  |  | Djukanovic et al 2013 |
| CRISPR | *MS8* | Knockout |  | Male sterility | No mutations recovered | Not reported | Not reported | Chen et al 2018 |
|  |  |  |  |  |  |  |  |  |
| CRISPR | *TMS5* | Knockout | Segregation of construct by self-fertilization | Male sterility | Thermosensitive male sterility | Not reported | No off-target mutation | Li et al 2017b |
| CRISPR | *MS33* | Knockout | Three gRNAs tested | Male sterility | Male sterility: absence of pollen grains in the three knockout  lines | Not reported | Not reported | Xie et al 2018 |
| CRISPR | *DMP* | Knockout and single-nucleotide replacement |  | Haploid inducer | Indel achieved marginal increase in haploid induction rate, single nucleotide mutation was more effective especially when combine with *mtl* mutation | Large increase in endosperm abortion rate | Not reported | Zhong et al 2019b |
| TALEN | *MTL* | Knockout |  | Haploid inducer | 6.7% haploid induction rate | Not reported | Not reported | Kelliher et al 2017 |
| CRISPR | *SMC3* | Knockout | All plants were mosaics | Defect in sister chromatid cohesion | Premature loss of sister chromatid cohesion but also defect in mitotic centromere pairing revealing new function | Not reported | Not reported | Zhang et al 2020a |
| CRISPR | *RF4* | Allele replacement | Used to create isogenic lines to test effect of substation F187Y | Fertility restoration | Plants containing allele F187 were  completely fertile and plants containing allele Y187 were sterile | Not reported | Not reported | Jaqueth et al 2020 |
| CRISPR | *GB1* | Knockout | Multiplex editing with two gRNAs | Developmental arrest | Seedling lethal (autoimmunity not developmental arrest) | Not reported | Not reported | Wu et al 2019 |
| CRISPR | *ZB7* in euchromatin, also 12 targets in heterochromatin | Indels | Demonstrated modification in euchromatin and heterochromatin | Albino plant (euchromatin) | Albino zb7 mutant was mosaic; heterozygotes were normal | Not reported | No off-target events | Feng et al 2016 |
| CRISPR | *PSY1* and 90 other targets | Knockout of PSY1 and indels in other targets | 76 of the targets began with G and 14 did not – much higher editing efficiency for targets beginning with G | Albino for PSY1, molecular test only for other targets | Albino for PSY1 | Not reported | No off-targets | Zhu et al 2016 |
| CRISPR | *ZB7, ZYP1* and *SMC3* | Knockout | *ZB7* biallelic mutation frequency = 66% | *ZB7* albino, chlorosis | *ZB7* albino, chlorosis | Poor survival for *ZB7* and *SMC3* | Not reported | Feng et al 2018 |
| CRISPR | Safe harbor sites in chromosomes 1, 3 and 10. | Insertion of target locus, followed by transgene insertion by homologous recombination | Concept to introduce a complex trait locus (CTL) comprising multiple linked sites for transgene insertion by homologous recombination, allowing stacking by conventional breeding | Dependent on integrated transgene. | Dependent on integrated transgene. | Not reported, but effects might arise depending on integrated transgenes | Not reported | Gao et al. 2020c |
| ZFN | Trait landing pads | Knockout and precise insertion | Proof of concept (gene stacking) | Molecular test only | Molecular test only | Not reported | Not reported | Kumar et al 2015 |
| CRISPR | Simplex editing: *MADS, MYBR* and *AP2* genes.  Multiplex editing: *RPL, PPR* and two lncRNA genes | Knockouts and single-base replacements | Simplex and multiplex editing, the latter based on the tRNA processing system | Molecular test only | Molecular test only | Not reported | Not reported | Qi et al 2016 |
| CRISPR | *AGO18A, AGO18B*, difydroflavonol 4‑reductase (*a1, a4*) | Knockout | Monoallelic and biallelic mutations in various combinations | Molecular test only | Molecular test only | Not reported | Not reported | Char et al 2017 |
| CRISPR | 20 genes (individual and families) | Knockout | 28 gRNAs expressed from 12 plasmids | Molecular test only | Molecular test only | Not reported | Not reported | Doll et al 2019 |
| MN I-SceI | Remobilization of a transgene by excision and subsequent use as donor for homologous recombination | Precise insertion | In-genome donor sequence generation | Molecular test only | Molecular test only | Not reported | Not reported | Ayar et al 2013 |
| TALEN vs CRISPR/Cas9 | *IPK1A, IPK* and *MRP4* | Knockout | Proof of concept and system comparison | Molecular test only | Molecular test only | Not reported | Not reported | Liang et al 2014 |
| CRISPR | *LG1, ALS2, MS26* and *MS45* | Knockout (*LG1*, *ALS2,* *MS26* and *MS45*) and allele replacement (*ALS2*) | DNA constructs and RNPs achieved similar mutation efficiencies | Molecular test only | Molecular test only | Not reported | Off-target mutations (target MS45) 0.18% with DNA and 0.01% with RNP | Svitashev et al 2016 |
| CRISPR | *MS26, MS45* and *LG1* | Knockout | The gRNA for MS45 was deliberately promiscuous to test for off-target mutations | Molecular test only | Molecular test only | Not reported | Off-target mutations only observed for promiscuous gRNA | Young et al 2020 |
| CRISPR/Cas9 nickase vs TALEN | *MEE1* | Knockout and insertion | Nickase achieved knockout by NHEJ indels and also larger deletions between DSBs. TALENs did not cause mutations. | Molecular test only | Molecular test only | Not reported | Cas9 nickase acts on methylated targets | Wolter et al 2017 |
| MN I-SceI | I-SceI sites | Indels | Transient activation of I‑SceI by crossing with plants expressing FLP recombinase | Molecular test only | Molecular test only | Not reported | No off-target events | Yang et al 2009 |
| CRISPR | Remobilization of a transgene by excision and subsequent use as donor for homologous recombination | Precise insertion | In-genome donor sequence generation, controlled by a heat-shock promoter | Molecular test only | Molecular test only | Not reported | Not reported | Barone et al 2020 |
