# Supplementary material for: Genome editing in cereal crops: an overview
Source: Transgenic Res. 2021 Jul 14;30(4):461–98. doi: 10.1007/s11248-021-00259-6 (PMC8316241; doi:10.1007/s11248-021-00259-6)
Supplement: Supplementary file 2 — Supplementary file2 (DOCX 46 KB) [file 11248_2021_259_MOESM2_ESM.docx]

Supplementary Table 2. Rice

| **Method^^[[1]](#footnote-1)^^** | **Target^^[[2]](#footnote-2)^^** | **KO/KI/PM^^[[3]](#footnote-3)^^** | **Notes** | **Anticipated phenotype** | **Achieved phenotype** | **Pleiotropic effects (morphology)** | **Pleiotropic effects (molecular)** | **Reference(s)** |
| --- | --- | --- | --- | --- | --- | --- | --- | --- |
| CRISPR | *OsGS3, Gn1a, OsGW2* | KO | Multiplex gene editing in J809 and L237 elite rice cultivars targeting for yield improvement | No. of tillers per plant, grain length, no. of grains per plant traits. | Average number of flowers per panicle in the triple mutants was much higher than the number in WTs. Grain length and width in the triple mutant increased about 2 mm (length) and 1.5 mm (width) | n/a | Total of 30 T0 plants were surveyed at  potential off-target sites with Sanger sequencing of the PCR  amplicon | Zhou et al  2018 |
| CRISPR | *Gn1a*, *DEP1, GS3, and IPA1* | KO | Each gene targeted at one site | *Gn1a*, enhanced grain number; *DEP1*, enhanced panicle size; *GS3*, enhanced grain size; *IPA1*, control tillering | The *gn1a*, *dep1*, and *gs3* mutants featured enhanced grain number (ca 2 fold), dense erect panicles, and larger grain size, respectively. The *ipa1* mutants showed two contrasting phenotypes, having either fewer tillers or more tillers, depending on the changes induced in the OsmiR156 target region | The size of the grain and the plant height were reduced in *dep1* mutant. Semi-dwarf, and grain with long awn, phenotypes were observed in *dep1* and *gs3* mutants, respectively | n/a | Li et al 2016 |
| CRISPR | *OsDEP, Gn1a(2x)* | KO | Targeted mutagenesis of *Gn1a* and *OsDEP1* gene, their sequences link in front of the sgRNA cassette designed in the fourth exon of *Gn1a* and the fifth exon of *OsDEP1* | Higher yield attributing traits, increased seed setting rate, 1000 grain weight, no. of panicles per plant and no. of grains per panicle. | Mutants showed significant increase in yield per plant | n/a | Insertion in two alleles and deletion in four alleles and one allele was inserted and six alleles were deleted | Huang et al  2018 |
| CRISPR | *GS3, Gn1a* | KO | Targeted mutagenesis of *GS3* and *Gn1a* gene to improve yield components of rice | No. of tillers, panicles and grains per plant | 7 of the 10 novel genotypes had decreased grain yields per plant as compared to the WT. Only 3genotype shad higher grain yields than the WT | Grain lengths of some mutants increased, and their grain volumes & grain weights were both slightly reduced compared with the WT | *GS3* and double *GS3-Gn1a* mutants were obtained | Shen et al  2018 |
| CRISPR | *PYL1, PYL4, PYL6* | KO | Multigene knockouts of rice *PYL* genes for studying yield enhancement | No of panicles per plant, no. of tillers per plant, 1000 grain weight like yield traits. | Mutants exhibited more primary and secondary  branches in the panicles along with higher spikelet number per main panicle than the WT | The single *pyl* mutants, only *pyl1* and *pyl12* exhibited significant defects in seed dormancy | n/a | Miao et al  2018 |
| CRISPR | *FWL genes, OsFWL1,*  *OsFWL4* | KO | Targeted mutagenesis of rice *FW 2.2*-like gene revealing *OsFWL4* as regulator for tiller no in plants. | No of tillers per plant, 1000 grain weight, no. of grains per main panicle, flag leaf width | The number of tillers, grain length per mutant plants along flag leaf width were greater than the WTs | n/a | Single-base mismatch with sgRNA exhibited  off target effects. Mutations were detected in four of the five and three of the ten loci | Gao et al  2020b |
| CRISPR | *OsPIN5b, GS3, OsMYB30* | KO | Two target sites of each gene were selected: *OsPIN5b*-site1 and *OsPIN5b*-site2, *GS3*-site1 and *GS3*-site2, and *OsMYB30-*site1 and OsMYB30-site2 | Yield attributing traits such as panicle height, no. of panicle per plant, pollen fertility and seed setting rate and cold tolerance | *OsPIN5b* mutants, the gs3 mutants, and the *OsMYB30* mutants exhibited increased panicle  length, enlarged grain size and increased cold tolerance respectively | Increase in panicle length due to increase in the auxin level in the *OsPIN5b* mutants | Six mutants had  off-target events at the putative site of *OsMYB30*-site1 | Zeng et al  2020c |
| CRISPR | *qSH1* | KO | Gene targeted at two sites | Reduced seed shattering | 69% increase in breaking tensile strength (negatively correlated with degree of shattering) | n/a | n/a | Sheng et al 2020 |
| CRISPR | *Rc* | Restore the function | Gene targeted at two sites, reverting the 14-bp frame-shift deletion to in-frame mutations (15-, 30-bp deletion) | Higher proanthocyanidin and cyanidin contents (pericarp colour) | Five-fold increase in proanthocyanidin, and 17-fold increase in cyanidin | n/a | n/a | Zhu et al 2019 |
| CRISPR | *F3′H, DFR,* and *LDOX* | KO | Three genes targeted with different gRNAs | Reduced total anthocyanin content (pericarp colour) | 91-94% reduction in total anthocyanin content | n/a | n/a | Jung et al 2019b |
| CRISPR | *NAC2* | KO | Gene targeted at one site | Characterization of *NAC2* | The roots of *OsNAC2-cas9* plants were longer primary roots and more crown roots than the WT roots. *OsNAC2-cas9* plants showed stronger root system than WT | n/a | n/a | Mao et al 2020 |
| CRISPR | *PIN1* | KO | Gene targeted at one site | Function analysis of four *PIN1* homologs (*OsPIN1a–1d*) | Compared with the WT, the *OsPIN1* single mutants had no dramatic phenotypes, but the *pin1a pin1b* double mutant had shorter shoots and primary  roots, fewer crown roots, longer root hairs and larger panicle branch angle Furthermore, the *pin1c pin1d* double mutant showed no observable phenotype at the seedling stage, but showed naked, pin-shape inflorescence at flowering | n/a | n/a | Li et al 2019b |
| CRISPR | *BU1* and *BC1* | KO | Two target sites of each gene | Reduced leaf inclination | Both of the *bu1* and *bc1* mutants displayed erected leaves | n/a | n/a | Ruan et al 2018 |
| CRISPR | *GA20ox2* | KO | Gene targeted at two sites | To establish CRISPR/Cas9 system that can be used in commercial cultivars for practical use of  genome editing in molecular breeding | Reduced plant height (22.2%), flag leaf length (FLL) and increased yield per plant (YPP) (6.0%) |  | Reduced gibberellins (GA) | Han et al 2019 |
| CRISPR | *SRL1* and *SRL2* | KO | Two target sites of each gene | To investigate the functional role of *SRL1* and *SRL2* | Homozygous mutants exhibit decreased chlorophyll content, transpiration rate, stomatal conductance, vascular bundles (VB), stomatal number, and agronomic traits with increased panicle number and bulliform cells (BCs). Under drought stress, mutant plants displayed lower malondialdehyde (MDA) content while higher survival rate, abscisic acid (ABA) content, superoxide dismutase (SOD), catalase (CAT) activities, and grain filling percentage compare with their wild type (WT) | n/a | n/a | Liao et al 2019 |
| CRISPR | *ACS1* and *ACS2* | KO | Each gene targeted at one site | Identification of roles of *ACS1* and *ACS2* in ethylene biosynthesis, in response to Pi deficiency | Lateral root elongation is decreased in *osacs1* and *osacs2* mutants under Pi-deficient conditions. Both  *osacs* mutants displayed reduced shoot and root lengths compared with the wild type (WT) in Pi-sufficient conditions, they still responded to Pi deficiency like the WT, resulting in shorter shoots and roots | n/a | Reduced ethylene production in the dark | Lee et al 2019 |
| CRISPR | *AUX3* | KO | Gene targeted at one site | Identification and validation of *AUX3* | Had shorter primary roots (PRs), decreased lateral root (LR) density, and longer root hairs (RHs) compared with their WT | n/a | n/a | Wang et al 2019b |
| CRISPR | *LPR5* | KO | Gene targeted at one site | Function analysis of *LPR5* | Significant reduction in the primary root length of the mutants compared with the WT after 4 d in both the +P and –P treatments. The height of the *oslpr5* mutants was significantly reduced compared with the WT, and they also exhibited significant reductions in seed-setting rate, 1000-grain weight, and grain yield per plant | n/a | n/a | Ai et al 2020 |
| CRISPR | *ABA1* | KO | Gene targeted at one site | A positive regulator of root growth and a negative regulator of plant height | A short root phenotype accompanied with a slender plant phenotype | All the mutant *ABA1* KO lines died in the field  without seed setting, indicating that ABA is essential for plants to complete their life cycle under ambient  conditions | n/a | Lin et al 2020a |
| CRISPR | *NIT1, NIT2, and NAR2.1* | KO | Each gene targeted at one site | Characterization of *NAR2.1* interaction with *NIT1* and *NIT2* | *osnit1* and *osnit2* displayed decreased primary root length and lateral root density. Double KO of *OsNAR2.1* and *OsNIT2* caused further decreases in lateral root density under nitrate supply | n/a | n/a | Song et al 2020 |
| CRISPR | *LC4* | KO |  | Characterization of *LC4* | Reduced leaf inclination | n/a | n/a | Qu et al 2019 |
| CRISPR | *PsbS1* and *PsbS2* | KO | Two target sites of each gene | *PsbS1,* decreased nonphotochemical quenching (NPQ) values; *PsbS2,* no effect on NPQ values | *OsPsbS1* KO mutant exhibited drastically decreased NPQ values. However, no significant differences were found in NPQ values between the *OsPsbS2* KO mutants and the WT plants | n/a | n/a | Wang et al 2017 |
| CRISPR | *Ghd7.1* | KO | Gene targeted at one site | A major QTL for both flag leaf width and length | Reduced leaf size | n/a | n/a | Tang et al 2018 |
| CRISPR | *NAL8* | KO | Gene targeted at one site | Identification of *NAL8* | Displayed a narrow flag leaf, reduced plant height, and fewer spikelets |  | The auxin levels in *nal8* mutants are higher than in the indica rice variety ¨TeQing¨ (TQ), while  the cytokinin levels are lower than in TQ | Chen et al 2019 |
| CRISPR | *qLL9* | KO | Gene targeted at one site | Identification of *qLL9* | The flag leaf length and area were significantly decreased | n/a | n/a | Fu et al 2019 |
| CRISPR | *RR21*, *RR22*, *RR23,* and *RR24* | KO | Each gene targeted at one site | Roles of *RRs* in growth, development and cytokinin signaling | *rr21/22/23* KO lines exhibited decreased height by 16% compared with WT, more tillers. The flag leaves of *rr21/22/23* averaged 77% of the length, 85% of the width and 65% of the area of the WT flag leaves. The *rr21/22/23* mutants exhibited a significant decrease in panicle length and in the number of primary and secondary branches, The effects of the *rr21/22/23* mutations on panicle architecture resulted in a significant decrease in the total number of spikelets per panicle | *rr24* KO lines were completely infertile and  were maintained in the hemizygous state for *rr24* | n/a | Worthen et al 2019 |
| CRISPR | *GRF7* | KO | Gene targeted at two sites | Larger angles of the leaf and taller plant | The angles of the second leaves of *GRF7KO-2*, *GRF7KO-14*, *GRF7KO-64*, and *GRF7KO-66* mutants were 27.6°, 26.1°, 32.9°, and 36.1°, respectively, which are significantly larger than those of the WT line (14.8°). The plant height of KO lines was significantly increased when compared with the WT | n/a | n/a | Chen et al 2020 |
| CRISPR | *GW2, GW5* and  *TGW6* | KO | Each gene targeted at one site | To improve grain weight | The T2 mutants exhibited significantly increased grain size and thousand-grain weight (TGW) compared with the WT. The double mutant exhibited increases of 11.69%, 8.47% and 12.68% in grain length, grain width and TGW, respectively; the four triple mutants displayed increases of 24.21%-25.32%, 19.79%-20.46% and 27.13%-29.84% in the grain length, grain width and TGW, respectively | n/a | n/a | Xu et al 2016 |
| CRISPR | *CCD7* | KO | Gene targeted at two sites | Reduced height and increased tillering | A striking increase in tillering, combined with a reduced height | n/a | n/a | Butt et al 2018 |
| CRISPR | *AAP3* | KO | Gene targeted at one site | Identification of *AAP3* | Tiller number, biomass of straw, grain yield were increased significantly | n/a | n/a | Lu et al 2018 |
| CRISPR | *LGG* | KO | Gene targeted at one site | Characterization of *LGG* | Led to longer grains than the WT | n/a | n/a | Chiou et al 2019 |
| CRISPR | *SD1* | KO | Gene targeted at one site | Shorter plant height | Shorter plant height for better resistance to lodging | n/a | n/a | Hu et al 2019a |
| CRISPR | *RGG2* | KO | Gene targeted at one site | Function analysis of *RGG2* | Exhibited enhanced growth, including elongated internodes, increased 1000-grain weight and plant biomass and enhanced grain yield per plant (+11.8% and 16.0%, respectively) | n/a | n/a | Miao et al 2019 |
| CRISPR | *AAP5* | KO | Gene targeted at two sites | Identification of *AAP5* | Increases in tiller number and grain yield | n/a | n/a | Wang et al 2019c |
| CRISPR | *LOGL5* | KO | Gene targeted at three sites | Function analysis of *LOGL5* | Resulted in normal rice plant morphology but with  increased grain yield under well-watered, drought, normal nitrogen, and low nitrogen field conditions at multiple geographical locations | n/a | n/a | Wang et al 2020c |
| CRISPR | *OsSWEET11, OsSWEET13, OsSWEET14* | PM | Multiplex CRISPR editing of effector binding elements (EBE) sites in *SWEET* promoters. | Broad and durable resistance to bacterial blight in rice | Resistance to the target region *Xoo* strains | Majority of lines have no pleiotropic effect, 1 line showed reduced fertility compared to wild type | n/a | Oliva et al 2019 |
| CRISPR | *OSSWEET11*  *OsSWEET 13*  *OsSWEET14* | PM | Multiple EBE alterations of the three *OsSWEET* genes | Broad spectrum resistance against bacterial blight in rice | Mutant line MS14K exhibited resistance to a large set of *Xoo* strains | Mutants show reduced plant height, normal tiller number and seed size | n/a | Xu et al 2019 |
| TALEN | *OsSWEET14* | PM | Double transgenic individuals that encompass all three EBEs (TalC, AvrXa7, Tal5/TalF) | Loss of bacterial blight disease susceptibility | Plants edited with AvrXa7 or Tal5 EBEs were resistant. Plants edited with TalC EBE did not confer resistance even with the reduction of *SWEET14* expression, suggesting other susceptibility gene(s) targeted by TalC effector | n/a | n/a | Blanvillain-Baufume et al 2017 |
| TALEN | *OsSWEET14* | PM | Pairs (2) of TALENs to induce mutations (independently deployed) in *OsSWEET14* EBE for AvrXa7 that overlaps with another EBE for PthXo3 | Loss of bacterial blight disease susceptibility by interfering with virulence function of AvrXa7 and PthXo3 | TALEN modified T2 plants resistant to AvrXa7 or PthXo3-dependent *Xoo* strains | n/a | n/a | Li et al 2012 |
| CRISPR | *OseIF4G* | KO | Three gRNAs designed | Resistance to Rice tungro spherical virus | T2 plants generated from event 1147 with substituions/deletions located immediate of YVV residues showed resistance to RTSV infection  Homozygous mutations in T0 plants resulting to truncated eIF4G rendered sterility | A T0 event with homozygous mutation with a five nucleotide deletion resulting to production of truncated eIF4G was sterile | Alleles in biallelic mutations are inherited at different frequencies resulting to distorted segregation pattern | Macovei et al 2018 |
| CRISPR | *OsERF922* | KO | One gene, multiple (2/3) target sites | Resistance to Blast | T2 homozygous mutant lines showing enhanced resistance to blast | n/a | T1 plants from chimeric KS-12 line and bi-allelic KS2-45 line did not follow Mendellian segregation ratio | Wang et al 2016 |
| CRISPR | *AHAS; ALS* | AR | Construct containing Cas9, two gRNAs, and donor repair template | Functional validation of candidate herbicide tolerance gene | AHAS-ΔW458 remained active at the presence of maximum herbicide concentration; Precise substitution of rwo discrete amino acid residues in ALS. | No effect in agronomic trait and yield | n/a | Fang et al 2020; Sun et al 2016 |
| CRISPR | *OsRR22* | KO | Target sequence having difference of at least two bases compared with similar non-target within PAM /PAM-proximal region | Higher salinity tolerance with no penalty in yield | Two mutant lines grew better than WTs in 75% NaCl solution. with better plant height and dry weight | n/a | Two homozygous, two heterozygous  mutations, and five bi-allelic mutations | Zhang et al 2019 |
| CRISPR | *OsDST* | KO | Two different gRNAs to target regions of DST protein | Enhanced leaf water  retention under dehydration stress developing both drought and salinity tolerance | Chlorophyll  retention was significantly higher in the excised leaves of the T_2_ mutant under osmotic and salt stresses and also with higher rate of lead elongation | Stomatal density decreased significantly in the T_1_ and T_2_  mutant plants, showing less water loss | Mutant  alleles of *OsDST* gene having  366 bp deletion between the two gRNAs | Santosh Kumar et al 2020 |
| CRISPR | *OsEPFL9* | KO | Targeted mutagenesis of the Exon1 of *OsEPFL9* | Improving water use efficiency with targeted mutagenesis | Stomatal density was improved in mutants. | n/a | n/a | Yin et al  2019 |
| CRISPR | *OsSRL1,*  *OsSRL2* | KO | CRISPR/Cas9-based mutagenesis of Semi-rolled leaf 1,2 (*SRL1* and *SRL2*) genes | Improving drought tolerance by developing essential leaf morphology | Hybrids  derived from mutant restorers showed semi-rolled leaf phenotype with increased panicle number,  grain number per panicle, and yield per plant | Mutant lines resulted decreased chlorophyll contents which also caused a decrease in Chl a/b compared to WTs | Total 19 positive mutant plants were obtained  including 19.3% homozygous and 37.5% heterozygous mutations | Liao et al  2019 |
| CRISPR | *OsNAC14* | KO | CRISPR/Cas9 based editing  targeting CDS region (700–722 bp) of *OsNAC14* gene | Functional validation of drought transcription factor | Reduced expression of downstream genes as expected. OX shows drought tolerance. | Decrease in panicle length and number of total grain compared to WTs | n/a | Shim et al  2018 |
| CRISPR | *OsSBEIIb* | KO | Gene targeted at one site (on the exon 12) with wild type Cas9 and single gRNA | Increase in amylose and resistant starch content | The total starch content in the mutant line was reduced by 26% and the amylose content increased from 19.6 to 27.4% of total starch. Resistant starch increased from 0.2 to 17.2 % | Produced opaque seeds. Increase in the accumulation of sugars, fatty acids, amino acids, and phytosterols | Increased the accumulation of multiple fatty acids, including C18:0 (1.3-fold), C14:0, C16:0, C18:2 and C22:0 (>1.5-fold), C20:0 (2.6-fold) and C15:0 increased from 0 to >2 µg/g. Also increased the accumulation of alanine, aspartic acid, glycine, lysine, proline, serine and valine which were not detectable in wild-type. Alanine, aspartic acid and proline all accumulated to >100 µg/g. Furthermore, the concentrations of the phytosterols campesterol, stigmasterol and β-sitosterol were >1.7-fold higher in the mutant | Baysal et al 2020 |
| CRISPR | *Cytosolic AGPase Large Subunit “APL2”*  *OsAPL2* | KO | Gene targeted at three sites with three sgRNAs.  For the wild-type Cas9, a single target site was in exon 13. For Cas9D10A nickase, two adjacent targets in the first exon | Reduction in starch content | Starch depletion in the leaves (85% reduction) | Increased soluble sugar in leaves 40 % | Cytosolic AGPase large subunit gene APL2 induce  the ectopic expression of APL2 and the corresponding small  subunit gene APS2b in rice leaves | Perez et al 2018 |
| CRISPR | *WAXY/GBSSI* | KO | Gene targeted at three sites | Reduction in amylose content | Amylose content declined to 8-12% in heterozygous seeds and to as low as 5% in homozygous seeds. Fully translucent seeds were obtained.  Mutations reduced but did not abolish GBSS activity in seeds due to partial compensation caused by the upregulation of GBSSII. The GBSS activity in the mutants was 61-71% of wild-type levels | Increased soluble sugar in seed 57 % | Modulation of starch biosynthetic pathways gene expression resulted in changes in AGPase and sucrose synthase activity. | Perez et al 2019 |
| CRISPR | *Plastidic phosphoglucomutase, OspPGM*  *Plastidic large subunit of ADP-glucose (ADP-Glc) pyrophosphorylase, OsAGPL4* | KO | Gene targeted at one site (on the fourth exon of *OsAGPL4)* | Total starch deficiency | Total reduction in starch synthesis in rice grains | Mutation of *OspPGM* or *OsAGPL4* effected polen development and caused male sterility | Total sugar increased (the level is not mentioned in the paper) | Lee et al 2016 |
| CRISPR | *OsSPS1* and *OsSPS11* | KO | Two genes targeted with single gRNA | Reduction in sucrose content | The knockout mutants of *OsSPS1* showed a 46% reduction in SPS activity in the leaves  Carbohydrate content. in the leaves and plant growth were not significantly different from those of wild-type plants.  Double knockout mutant of *OsSPS1* and *OsSPS11* (*sps1/sps11*), reduced 84% leaf SPS activity and resulted in higher starch accumulation in the leaves than in the wild-type leaves | Higher starch accumulation in leaves. | A 50% reduction in SPS activity did not affect sucrose/starch ratio in rice leaves.  An 84% reduction in SPS activity caused increase in starch content in rice leaves.  An 84% reduction in SPS activity did not inhibit normal plant growth in rice | Hashida et al 2016 |
| CRISPR | *OsSWEET11* | KO | Gene targeted at three sites | Reduction in sucrose content | The knockout of *OsSWEET11* significantly decreased the sucrose concentration (40 %) in the mutant embryo sacs and led to defective grain filling compared with that of the wild-type (WT) plant | The panicles of the mutants are much lighter than those of the WT rice due to defective grain filling  and seed maturation was also delayed slightly in the mutant lines | Reduction in starch content in mature caryopses 5% | Ma et al 2017 |
| CRISPR | *OsINV2* and *OsINV3* | KO | Gene targeted with two gRNA (on the first exon of *OsINV3* and the third exon of *OsINV2)* | Altered sugar content | Loss-of-function of *OsINV3* decreased grain weight (33.5%).  *OsINV2*, that has no effect on grain size by itself  Increased sucrose and decreased hexose levels | Changes vacuolar invertase activities and starch constitution. | Amylose content decreased 3-6 %. | Deng et al 2020 |
| CRISPR | *Fatty acid desaturase 2 (OsFAD2)* | KO | *OsFAD2-1* gene targeted since it is highly expressed in rice seed compared with *OsFAD2-2* and *OsFAD2-3* | Increase in oleic acid content | 2-fold increase in oleic acid content | n/a | Decrease in linoleic acid content to undetectable levels | Abe et al 2018 |
| CRISPR | *OsPLDa1* | KO | The *OsPLDa1* gene has two transcripts with three and four exons, respectively. sgRNAs targeted the exon common to both transcripts | Reduction in myoinositol or phytic acid in rice seed. | Phytic acid reduced 9.89%, total phosphorus content reduced 10.5%. | 44 or 45 of 93-97 negative metabolites down-regulated, 63 of 160-162 positive metabolites up-regulated | Transcripts of *OsPLDα3*, *OsPLDα5*, and *OsPLDd1* increased 1.75, 7.2 and 13.2 fold, *PLDβ1* decreased 0.8 fold | Khan et al 2019 |
| CRISPR | *OsASN1* | KO | Gene targeted with two gRNAs | Reductio in synthesis of Asn in rice root | The concentration of Asn reduced to 50% in root and shoot, Glu reduced to 50% in shoot and 20% in root, Asp reduced to 80% in shoot and 15% in root, Gln increased 4-fold in root and 2-fold in shoot | Plant height reduced 33% and tiller number reduced 50% | Biomass reduced 50%, N influx rate per unit weight no change | Luo et al 2018 |
| CRISPR | *OsGAD3.* | KO | Gene targeted with two gRNAsa and KO. induced a 122 bp deletion in the *CaMBD*-coding region of *OsGAD3* | Increase GABA levels in rice seed | 7-fold higher content of GABA | 12% seed weight per grain and 20% protein content increase | Ser and Glu 1.6-fold increased, Ala, Asp, Met and Phe more than 2-fold increased, Leu 3.1-fold increased and Ile 5.2-fold increased.  Asn and Trp 0.3 fold decreased | [Akama](https://www.ncbi.nlm.nih.gov/pubmed/?term=Akama%20K%5bAuthor%5d&cauthor=true&cauthor_uid=32180062) et al 2020 |
| CRISPR | *OsCYP97A4, OsDSM2, OsCCD4a, OsCCD4b* and *OsCCD7* | KO | Each gene targeted at one site | Increase in β-carotene accumulation in rice endosperm | No significant change | Dwarf with more tillers and lower seed-setting | The biosynthesis of strigolactone is defective | Yang et al 2017 |
| CRISPR | *OsOr* | KO | Three gRNAs targeted different regions in the third and fourth intron of *OsOr.* | Accumulate β-carotene in rice callus | From below the detection limit increase to 2.7 ug/g fresh weight | n/a | n/a | Endo et al 2019 |
| CRISPR | *CYP75B3* and *CYP75B4* | KO | The target site of single knockout of *CYP75B3* was in first exon. The targets sites of double knockout of *CYP75B3* and *CYP75B* were in second exon | Accumulation of apigenin | cyp75b3-ko: apigenin increased 74% and tricin also increased 14%  cyp75b3and75b4-ko: apigenin increased from 0 to 108, and tricin is decreased from 40 to 0 (g^-1^, FW) | No change in growth phenotype | n/a | Lam et al 2019 |
| TALEN | *OsBADH2* | KO | The TALEN pair targeted the fourth exon of *OsBADH2* | Synthesis of 2-acetyl-1-pyrroline (2AP) in nonfragrant rice | 2AP content increased from 0 to 0.35-0.75 mg/kg | n/a | n/a | Shan et al 2015 |
| CRISPR | *OsCAld5H1* | KO | Three gRNAs targeted different regions of the first exon of *OsCAld5H1* | Increase in guaiacyl (G) and reduction in syringyl (S) | In culm G lignin unit increased from 44% to 65%; in leaf sheath G lignin unit increased from 79% to 96% | Panicle weight reduced 58%, spikelet fertility reduced 56% and culm length reduced 15% | Arabinan, xylan and galactan increased 28%, 56% and 18% respectively | Takeda et al 2019 |
| CRISPR | *OsSND2* | KO | Two target sites were 76-bp apart from each other in the first exon of *OsSND2* | Reduction in cellulose content in rice internodes and panicles | Cellulose content reduced from 340 to 280 (mg/g) | A little early flowering, thinner walls of sclerenchyma cell | *MYB61* and *CESA4, 7, 9* expression levels down-regulated appr. 50%  *MYB86L*, *MYB61L* and *OsMYB58/63* expression levels down-regulated 80% | Ye et al 2018 |
| CRISPR | *OsNramp5* | KO | Targeted mutagenesis *OsNramp5* for lower Cd accumulation in Rice | Grain Cd concentration was relatively low than the WTs | Cd concentration in grains (brown rice) was lower in the mutants <0.05 mg/kg,  compared with an average of 0.33 mg/kg and 2.90 mg/kg in the grains of WT plants in field levels | n/a | Four types of mutation found - homozygotes with both alleles,  heterozygotes with only one allele mutated,  bi-allelic mutants with both copies of the target | Tang et al 2017 |
| CRISPR | *OsLCT1, OsNramp5* | KO | Generation of mutant lines for breeding for low Cd rice. | No. of tillers per plant, no. of grains per plant, Cd concentration in grains. | *OsNramp5* mutants retained a low Cd conc. in the brown rice grains, i.e., 0.04-0.06mg/kg | Stunted plant growth of the mutants | The 33-nt deletion would only result in a loss of 11 a.a. of the *OsNramp5* | Songmei et al 2019 |
| CRISPR | *OsNramp5* | KO | Targeted mutagenesis for low Cd accumulation in rice | No. of panicles per plant, root and shoot dry weight, chlorophyll content and Cd concentration in grains | The mutants had lower Cd concentration in both  roots and shoots at all Mn concentrations. Number of panicles  per plant was significantly increased in mutants | Plant height was significantly reduced in all three mutant alleles compared to  their wild-type plants. Both seed setting rate and total seed number  per panicle were decreased in the mutants | 1-bp deletion in first exon of *OsNRAMP5*,  *osnramp5-5* and *osnramp5-6* having17- and 11-bp deletions in the first exon, respectively | Yang et al 2019 |
| CRISPR | *SSU-crtI* and *ZmPsy30* | KI | Targeted insertion of a 5.2 kb marker-free DNA fragment at 2 genomic safe harbors. | Obtain carotenoid-enriched rice. | Color of seed is golden, β-carotene content increased from 0 to 7.90 (μg g^−1^, DW) | No change in plant height and grain dimensions | n/a | Dong et al 2020 |
| CRISPR | *NRT1.1B* | AR | Construct containing Cas9, two gRNAs, and donor repair template | Allele replacement proof of concept by targeting gene for iproved nitrogen use efficiency | n/a | n/a | *n/a* | Li et al 2018 |
| ZFN | *SSIVa* | KO | ZFN-mediated gene disruption | Elucidation of SSIVa gene function | No SSIVa mRNA expression in transgenic plants with premature stop codon and substitution events | No effect on other starch synthesis related genes | n/a | Jung et al 2017 |

1. TALEN = transcription activator-like effector nucleases, CRISPR = clustered regularly interspaced short palindromic repeats [↑](#footnote-ref-1)
2. Target gene [↑](#footnote-ref-2)
3. KO = knockout, KI = knock-in, PM = promoter modification [↑](#footnote-ref-3)
