# Supplementary material for: Genome editing in cereal crops: an overview
Source: Transgenic Res. 2021 Jul 14;30(4):461–98. doi: 10.1007/s11248-021-00259-6 (PMC8316241; doi:10.1007/s11248-021-00259-6)
Supplement: Supplementary file 3 — Supplementary file3 (DOCX 20 KB) [file 11248_2021_259_MOESM3_ESM.docx]

Supplementary Table 3. Wheat and barley

| **Method^^[[1]](#footnote-1)^^** | **Target^^[[2]](#footnote-2)^^** | **KO/KI/PM^^[[3]](#footnote-3)^^** | **Notes** | **Anticipated phenotype** | **Achieved phenotype** | **Pleiotropic effects (morphology)** | **Pleiotropic effects (molecular)** | **Reference(s)** |
| --- | --- | --- | --- | --- | --- | --- | --- | --- |
| CRISPR | *TaGW2, TaLpx-1 TaMLO* | KO | Simultaneous targeting using the polycistronic tRNA-gRNA system | n/a | Transgenerational activity, a T2 individual with a fixed edited *TaLptx-1* allele at homeolog B could be identified | n/a | n/a | Wang et al 2018b |
| CRISPR | *TaGW2 and TaGW7* | KO | Contribution of each of the *TaGW2* homeologs to the quantitative trait was investigated in detail | Altered yield, grain morphology and weight | Editing the *TaGW7* B and D homeologs led to increased grain width and weight but reduced grain length, while seeds of a triple mutant in *TaGW2* have increased TGW, grain area, width and length | n/a | n/a | Wang et al 2018a; Wang et al 2019; Wang et al 2018b |
| CRISPR | *Cytokinin oxidase/dehydrogenase (CKX)* | KO |  | Higher cytokinin accumulation and a higher number of reproductive organs | Increased grain number per spikelet | n/a | n/a | Zhang et al 2019a |
| CRISPR | *Qsd1* | KO | Homozygous, Cas-free mutants in all three homeologs were obtained | Prevention of pre-harvest sprouting | Homozygous mutants in all three homeologs germinated with a delay of about 5 days | n/a | n/a | Abe et al 2019 |
| CRISPR | *Ms1, Ms45* | KO | Study investigates the relevance and contribution of the different genes to trait | Male sterility to be used in hybrid seed production programs. | Homozygous individuals were shown to be male sterile | n/a | n/a | Okada et al 2019; Singh et al 2018 |
| TALEN | *TaMLO* | KO | Three homeologs of *MLO* present in the three subgenomes | Resistance to powdery mildew | Partial resistance upon pathogen attack | No unwanted pleiotropic phenotypes with respect to callose deposition | n/a | Wang et al 2014; Gruner et al 2020 |
| CRISPR | *NFXL1* | KO | Three pairs of homeologs were targeted using two separate U6 promoter-driven gRNAs complementary to two different regions present in all *NFLX1* copies | Partial *Fusarium* resistance (*NFXL1* downregulation via RNAi in barley confers partial *Fusarium* resistance) | Increased *Fusarium* head blight resistance, confirming a role of *NFXL1* in wheat *Fusarium* resistance | n/a | n/a | Brauer et al 2020 |
| CRISPR | *Edr1* | KO | Use of one sgRNA targeting all three homeologs resulted in T0 plants with simultaneously edited alleles | Mildew resistance | Reduced sensitivity to *Blumeria graminis* infection and reduced cell death | Phenotypic analyses of mutants in barley show no compromised plant growth parameters | n/a | Zhang et al 2017 |
| CRISPR | *Gliadin genes* | KO | Gliadin genes are encoded as multigene-families on all three subgenomes. Two sgRNAs were designed to target a conserved region adjacent to the immunodominant peptide sequence | 45 potential target genes were identified | Up to 35 different genes were mutated, immunoreactivity was reduced by 85% | n/a | n/a | Sánchez-León et al 2018 |
| CRISPR | *alpha- and gamma-gliadin* | KO | Multiplexing approach involving five separate gRNAs, two targeting alpha- and three gamma-gliadin genes | Comparison with results obtained with random mutagenesis | Gliadin profile alterations | n/a | n/a | Jouanin et al 2019 |
| CRISPR | *WTAI-CM3 and WTAI-CM16 (alpha-amylase/trypsin inhibitors)* | KO | Multiplexing approach using the polycistronic tRNA-gRNA system in *T. durum* | Reduction of allergenicity | Reduction of allergen content | n/a | Activation of the ATI 0.28 pseudogene | Camerlengo et al 2020 |
| CRISPR | *TaGW2* | KO | Investigates functional difference between and additive interaction  of *TaGW2* homoeologs | Increase in grain protein content | Positive effect on flour protein content and gluten strength | n/a | n/a | Zhang et al 2018 |

1. TALEN = transcription activator-like effector nucleases, CRISPR = clustered regularly interspaced short palindromic repeats [↑](#footnote-ref-1)
2. Target gene [↑](#footnote-ref-2)
3. KO = knockout, KI = knock-in, PM = promoter modification [↑](#footnote-ref-3)
